# Erratum: OncoScore: a novel, Internet-based tool to assess the oncogenic potential of genes

**DOI:** 10.1038/srep46823

**Published:** 2017-05-22

**Authors:** Rocco Piazza, Daniele Ramazzotti, Roberta Spinelli, Alessandra Pirola, Luca De Sano, Pierangelo Ferrari, Vera Magistroni, Nicoletta Cordani, Nitesh Sharma, Carlo Gambacorti-Passerini

Scientific Reports 7: Article number: 4629010.1038/srep46290; published online: 04 07 2017; updated: 05 22 2017

The original version of this Article incorrectly listed all author names in reverse. The author list now reads:

Rocco Piazza, Daniele Ramazzotti, Roberta Spinelli, Alessandra Pirola, Luca De Sano, Pierangelo Ferrari, Vera Magistroni, Nicoletta Cordani, Nitesh Sharma & Carlo Gambacorti-Passerini.

The ‘How to Cite’ section was therefore incorrect, and now reads:

Piazza, R. *et al*. OncoScore: a novel, Internet-based tool to assess the oncogenic potential of genes. Sci. Rep. **7**, 46290; doi: 10.1038/srep46290 (2017).

In addition, there were typographical errors in affiliations 3, 4 and 5 which now read:

Affiliation 3

GalSeq s.r.l., via Italia 46, Monza, 20900, Italy.

Affiliation 4

University of Milano-Bicocca, Dept. of Informatics, Milano, 20125, Italy.

Affiliation 5

University of New Mexico, Department of Pediatrics, Albuquerque, US.

Finally, the Acknowledgments section of this Article was incomplete, where:

“We kindly acknowledge the contribution of Michela Viltadi for technical help. This work was supported by Associazione Italiana Ricerca sul Cancro 2013 (IG-14249 to C.G.P.), by Associazione Italiana Ricerca sul Cancro 2015 (IG-17727 to R.P.), by Fondazione Berlucchi (2014) and by European Union’s Horizon 2020 Marie Skłodowska-Curie Innovative Training Networks (ITN-ETN) under grant agreement No.: 675712CGP; CGP is a member of the European Research Initiative for ALK-Related Malignancies (www.erialcl.net)”.

Now reads:

“We kindly acknowledge the contribution of Michela Viltadi for technical help. This work was supported by Associazione Italiana Ricerca sul Cancro 2013 (IG-14249 to C.G.P.), by Associazione Italiana Ricerca sul Cancro 2015 (IG-17727 to R.P.), by Fondazione Berlucchi (2014) and by European Union’s Horizon 2020 Marie Skłodowska-Curie Innovative Training Networks (ITN-ETN) under grant agreement No.: 675712CGP; CGP is a member of the European Research Initiative for ALK-Related Malignancies (www.erialcl.net). N.C. was supported by a Fondazione Umberto Veronesi fellowship 2015”.

These errors have now been corrected in the HTML and PDF versions of this Article.

