# Supplementary material for: OncoScore: a novel, Internet-based tool to assess the oncogenic potential of genes
Source: Sci Rep. 2017 Apr 7;7:46290. doi: 10.1038/srep46290 (PMC5384236; doi:10.1038/srep46290)
Supplement: Supplementary Data [file srep46290-s1.pdf]

OncoScore: a novel, Internet-based tool to assess the oncogenic potential of genes

Authors:

Piazza Rocco[1], Ramazzotti Daniele[2], Spinelli Roberta[1], Pirola Alessandra[3], De Sano Luca[4], Ferrari Pierangelo[3], Magistroni Vera[1], Cordani Nicoletta[1], Sharma Nitesh[5], Gambacorti-Passerini Carlo[1]

1) University of Milano-Bicocca, Dept. of Medicine and Surgery, Monza, 20900, Italy

2) Stanford University, Dept. of Pathology, California 94305, USA

3) GalSeq s.r.l., viale Italia 46, Monza, 20900, Italy

4) University of Milano-Bicocca, Dept. of Informatics, 20125, Milano

5) University of New Mexico, Department of Pediatrics, Albuquerque

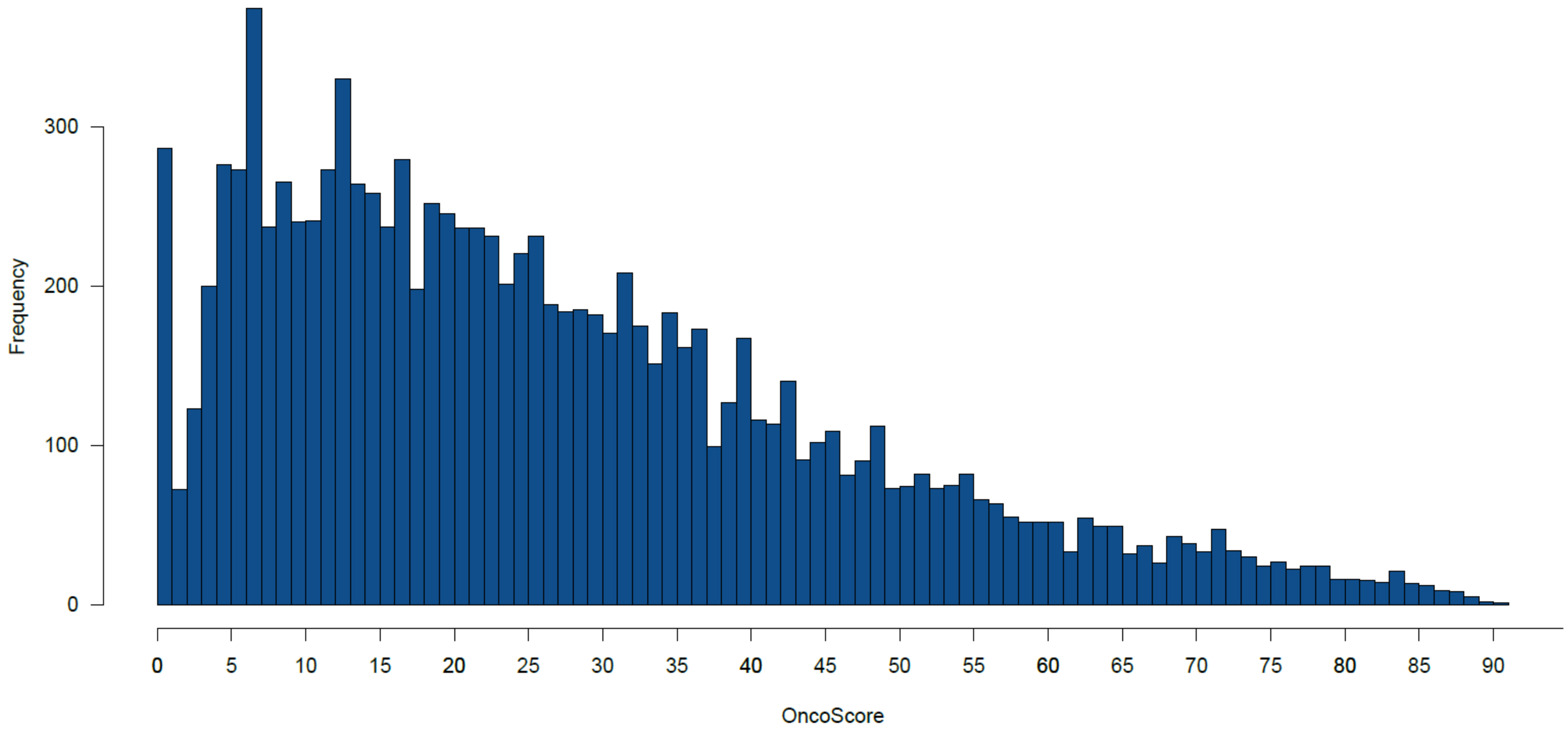

Suppl. Fig. 1. Distribution of the OncoScore across all the genes with more than 10 citations as of January 2016.

Suppl. Tab. 1. List of all the cancer genes comprised in the Cancer Gene Census dataset.

Symbol	Chr	Chr Band	Cancer Somatic Mut	Cancer Germline Mut	Tumor Types (Somatic)	Tumor Types (Germline)	Cancer Syndrome	Tissue Type	Cancer Molecular Genetics	Mutation Type	Translocation Partner
ABL1	9	9q34.1	yes		CML, ALL, T-ALL			L	Dom	T, Mis	BCR, ETV6, NUP214
ABL2	1	1q24-q25	yes		AML			L	Dom	T	ETV6
ACSL3	2	2q36	yes		prostate			E	Dom	T	ETV1
AF15Q14	15	15q14	yes		AML			L	Dom	T	MLL
AF1Q	1	1q21	yes		ALL			L	Dom	T	MLL
AF3p21	3	3p21	yes		ALL			L	Dom	T	MLL
AF5q31	5	5q31	yes		ALL			L	Dom	T	MLL
AKAP9	7	7q21-q22	yes		papillary thyroid			E	Dom	T	BRAF
AKT1	14	14q32.32	yes		breast, colorectal, ovarian, NSCLC			E	Dom	Mis	
AKT2	19	19q13.1-q13.2	yes		ovarian, pancreatic			E	Dom	A	
ALDH2	12	12q24.2	yes		leiomyoma			M	Dom	T	HMGA2
ALK	2	2p23	yes	yes	ALCL, NSCLC, neuroblastoma	neuroblastoma	familial neuroblastoma	L, E, M	Dom	T, Mis, A	NPM1, TPM3, TFG, TPM4, ATIC, CLTC, MSN, ALO17, CARS, EML4, KIF5B,

											C2orf22
ALO17	17	17q25<br>.3	yes		ALCL			L	Dom	T	ALK
APC	5	5q21	yes	yes	colorectal,<br>pancreatic<br>, desmoid,<br>hepatobla<br>stoma,<br>glioma,<br>other CNS	colorectal,<br>pancreatic<br>, desmoid,<br>hepatobla<br>stoma,<br>glioma,<br>other CNS	adenomat<br>ous<br>polyposis<br>coli;<br>Turcot<br>syndrome	E, M, O	Rec	D, Mis,<br>N, F, S	
ARHGE<br>F12	11	11q23<br>.3	yes		AML			L	Dom	T	MLL
ARHH	4	4p13	yes		NHL			L	Dom	T	BCL6
ARID1A	1	1p35.<br>3	yes		clear cell<br>ovarian<br>carcinoma<br>, RCC			E	Rec	Mis, N,<br>F, S, D	
ARID2	12	12q12	yes		hepatocell<br>ular<br>carcinoma			E	Rec	N, S, F	
ARNT	1	1q21	yes		AML			L	Dom	T	ETV6
ASPSC<br>R1	17	17q25	yes		alveolar<br>soft part<br>sarcoma			M	Dom	T	TFE3
ASXL1	20	20q11<br>.1	yes		MDS,<br>CMML			L	Rec	F, N, Mis	

ATF1	12	12q13	yes		malignant melanoma of soft parts, angiomatoid fibrous histiocytoma			E, M	Dom	T	EWSR1, FUS
ATIC	2	2q35	yes		ALCL			L	Dom	T	ALK
ATM	11	11q22.3	yes	yes	T-PLL	leukemia, lymphoma, medulloblastoma, glioma	ataxia-telangiectasia	L, O	Rec	D, Mis, N, F, S	
ATRX	X	Xq21.1	yes		Pancreatic neuroendocrine tumors, paediatric GBM			E	Rec	Mis, F, N	
AXIN1	16	16p13.3	yes	yes	colorectal, endometrial, prostate and hepatocellular carcinomas, hepatoblastoma, sporadic medulloblastoma			E, O	Rec	D, Mis, N, F, S	

BAP1	3	3p21.31-p21.2	yes		uveal melanoma, breast, NSCLC, RCC	mesothelioma, uveal melanoma		E	Rec	N, Mis, F, S, O	
BCL10	1	1p22	yes		MALT			L	Dom	T	IGH@
BCL11A	2	2p13	yes		B-CLL			L	Dom	T	IGH@
BCL11B	14	14q32.1	yes		T-ALL			L	Dom	T	TLX3
BCL2	18	18q21.3	yes		NHL, CLL			L	Dom	T	IGH@
BCL3	19	19q13	yes		CLL			L	Dom	T	IGH@
BCL5	17	17q22	yes		CLL			L	Dom	T	MYC
BCL6	3	3q27	yes		NHL, CLL			L	Dom	T, Mis	IG loci, ZNFN1A1, LCP1, PIM1, TFRC, CIITA, NACA, HSPCB, HSPCA, HIST1H4I, IL21R, POU2AF1, ARHH, EIF4A2, SFRS3
BCL7A	12	12q24.1	yes		BNHL			L	Dom	T	MYC
BCL9	1	1q21	yes		B-ALL			L	Dom	T	IGH@, IGL@
BCOR	X	Xp11.4	yes		retinoblastoma, AML, APL (translocation)				Rec	F, N, S, T	RARA
BCR	22	22q11.21	yes		CML, ALL, AML			L	Dom	T	ABL1, FGFR1, JAK2

BHD	17	17p11.2		yes		renal, fibrofolliculomas, trichodiscomas	Birt-Hogg-Dube syndrome	E, M	Rec?	Mis. N, F	
BIRC3	11	11q22-q23	yes		MALT			L	Dom	T	MALT1
BLM	15	15q26.1		yes		leukemia, lymphoma, skin squamous cell, other tumor types	Bloom syndrome	L, E	Rec	Mis, N, F	
BMPR1A	10	10q22.3		yes		gastrointestinal polyps	juvenile polyposis	E	Rec	Mis, N, F	
BRAF	7	7q34	yes		melanoma, colorectal, papillary thyroid, borderline ovarian, NSCLC, cholangiocarcinoma, pilocytic astrocytoma			E	Dom	Mis, T, O	AKAP9, KIAA1549
BRCA1	17	17q21	yes	yes	ovarian	breast, ovarian	hereditary breast/ovarian cancer	E	Rec	D, Mis, N, F, S	

BRCA2	13	13q12	yes	yes	breast, ovarian, pancreatic	breast, ovarian, pancreatic, leukemia (FANCB, FANCD1)	hereditary breast/ovarian cancer	L, E	Rec	D, Mis, N, F, S	
BRD3	9	9q34	yes		lethal midline carcinoma of young people			E	Dom	T	C15orf55
BRD4	19	19p13.1	yes		lethal midline carcinoma of young people			E	Dom	T	C15orf55
BRIP1	17	17q22		yes		AML, leukemia, breast	Fanconi anaemia J, breast cancer susceptiblity	L, E	Rec	F, N, Mis	
BTG1	12	12q22	yes		BCLL			L	Dom	T	MYC
BUB1B	15	15q15		yes		rhabdomyosarcoma	mosaic variegated aneuploidy	M	Rec	Mis, N, F, S	
C12orf9	12	12q14.3	yes		lipoma			M	Dom	T	LPP
C15orf21	15	15q21.1	yes		prostate			E	Dom	T	ETV1
C15orf55	15	15q14	yes		lethal midline carcinoma			E	Dom	T	BRD3, BRD4
C16orf75	16	16p13.13	yes		PMBL, Hodgkin lymphoma			L	Dom	T	CIITA

C2orf44	2	2p23.3	yes		NSCLC			E	Dom	T	ALK
CAMTA1	1	1p36.31-p36.23	yes		epithelioid haemangioendothelioma			M	Dom	T	WWTR1
CANT1	17	17q25	yes		prostate			E	Dom	T	ETV4
CARD11	7	7p22	yes		DLBCL			L	Dom	Mis	
CARS	11	11p15.5	yes		ALCL			L	Dom	T	ALK
CBFA2T1	8	8q22	yes		AML			L	Dom	T	MLL, RUNX1
CBFA2T3	16	16q24	yes		AML			L	Dom	T	RUNX1
CBFB	16	16q22	yes		AML			L	Dom	T	MYH11
CBL	11	11q23.3	yes		AML, JMML, MDS			L	Dom, Rec	T, Mis S, O	MLL
CBLB	3	3q13.11	yes		AML			L	Rec	Mis S	
CBLC	19	19q13.2	yes		AML			L	Rec	M	
CCDC6	10	10q21	yes		NSCLC			E	Dom	T	RET
CCNB1IP1	14	14q11.2	yes		leiomyoma			M	Dom	T	HMGA2
CCND1	11	11q13	yes		CLL, B-ALL, breast			L, E	Dom	T	IGH@, FSTL3
CCND2	12	12p13	yes		NHL,CLL			L	Dom	T	IGL@
CCND3	6	6p21	yes		MM			L	Dom	T	IGH@
CCNE1	19	19q12	yes		serous ovarian			E	Dom	A	
CD273	9	9p24.2	yes		PMBL, Hodgkin lymphoma			L	Dom	T	CIITA

CD274	9	9p24	yes		PMBL, Hodgkin lymphoma			L	Dom	T	CIITA
CD74	5	5q32	yes		NSCLC			E	Dom	T	ROS1
CD79A	19	19q13.2	yes		DLBCL			L	Dom	O, S	
CD79B	17	17q23	yes		DLBCL			L	Dom	Mis, O	
CDH1	16	16q22.1	yes	yes	lobular breast, gastric	gastric	familial gastric carcinoma	E	Rec	Mis, N, F, S	
CDH11	16	16q22.1	yes		aneurysmal bone cyst			M	Dom	T	USP6
CDK12	17	17q12	yes		serous ovarian			E	Rec	Mis, N, F	
CDK4	12	12q14		yes		melanoma	familial malignant melanoma	E	Dom	Mis	
CDK6	7	7q21-q22	yes		ALL			L	Dom	T	MLLT10
CDKN2A	9	9p21	yes	yes	melanoma, multiple other tumor types	melanoma, pancreatic	familial malignant melanoma	L, E, M, O	Rec	D, Mis, N, F, S	
CDKN2a (p14)	9	9p21	yes	yes	melanoma, multiple other tumor types	melanoma, pancreatic	familial malignant melanoma	L, E, M, O	Rec	D, S	
CDKN2C	1	1p32	yes		glioma, MM			O, L	Rec	D	
CDX2	13	13q12.3	yes		AML			L	Dom	T	ETV6
CEBPA	19	19q13.1	yes		AML, MDS			L	Dom	Mis, N, F	
CEP1	9	9q33	yes		MPD, NHL			L	Dom	T	FGFR1

CHCHD7	8	8q11.2	yes		salivary gland adenoma			E	Dom	T	PLAG1
CHEK2	22	22q12.1		yes		breast	familial breast cancer	E	Rec	F	
CHIC2	4	4q11-q12	yes		AML			L	Dom	T	ETV6
CHN1	2	2q31-q32.1	yes		extraskeletal myxoid chondrosarcoma			M	Dom	T	TAF15
CIC	19	19q13.2	yes		oligodendroglioma, soft tissue sarcoma			O	Rec	Mis, F, S,T	DUX4
CIITA	16	16p13	yes		PMBL, Hodgkin lymphoma			L	Dom	T	FLJ27352, CD274, CD273, RALGDS, RUNDC2A, C16orf75, BCL6
CLTC	17	17q11-qter	yes		ALCL, renal			L	Dom	T	ALK, TFE3
CLTCL1	22	22q11.21	yes		ALCL			L	Dom	T	?
CMKOR1	2	2q37.3	yes		lipoma			M	Dom	T	HMGA2
CNOT3	19	19q13.4	yes		T-ALL			L	Dom	Mis, N	
COL1A1	17	17q21.31-q22	yes		dermatofibrosarcoma protuberans, aneurysmal bone cyst			M	Dom	T	PDGFB, USP6

COPEB	10	10p15	yes		prostate, glioma			E, O	Rec	Mis, N		
COX6C	8	8q22-q23	yes		uterine leiomyoma			M	Dom	T	HMGA2	
CREB1	2	2q34	yes		clear cell sarcoma, angiomatoid fibrous histiocytoma			M	Dom	T	EWSR1	
CREB3L1	11	11p11.2	yes		myxofibrosarcoma			M	Dom	T	FUS	
CREB3L2	7	7q34	yes		fibromyxoid sarcoma			M	Dom	T	FUS	
CREBBP	16	16p13.3	yes		ALL, AML, DLBCL, B-NHL			L	Dom/Rec	T, N, F, Mis, O	MLL, MORF, RUNXBP2	
CRLF2	X,Y	Xp22.3; Yp11.3	yes		B-ALL, Downs associated ALL			L	Dom	Mis, T	P2RY8, IGH@	
CRTC3	15	15q26.1	yes		salivary gland mucoepidermoid			E	Dom	T	MAML2	
CTNNB1	3	3p22-p21.3	yes		colorectal, ovarian, hepatoblastoma, pleomorphic salivary gland adenoma, other tumor types			E, M, O	Dom	H, Mis, T	PLAG1	

CYLD	16	16q12-q13	yes	yes	cylindroma	cylindroma	familial cylindromatosis	E	Rec	Mis, N, F, S	
D10S170	10	10q21	yes		papillary thyroid, CML			E	Dom	T	RET, PDGFRB
DAXX	6	6p21.3	yes		pancreatic neuroendocrine tumor, paediatric glioblastoma			E	Rec	Mis, F, N	
DDB2	11	11p12		yes		skin basal cell, skin squamous cell, melanoma	xeroderma pigmentosum (E)	E	Rec	Mis, N	
DDIT3	12	12q13.1-q13.2	yes		liposarcoma			M	Dom	T	FUS
DDX10	11	11q22-q23	yes		AML*			L	Dom	T	NUP98
DDX5	17	17q21	yes		prostate			E	Dom	T	ETV4
DDX6	11	11q23.3	yes		B-NHL			L	Dom	T	IGH@
DEK	6	6p23	yes		AML			L	Dom	T	NUP214
DICER1	14	14q32.13	yes	yes	sex cord-stromal tumor, TGCT, embryonal rhabdomyosarcoma	pleuropulmonary blastoma	familial pleuropulmonary blastoma	E, M, O	Rec	Mis F, N	
DNM2	19	19p13.2	yes		ETP ALL			L	Rec	F, N, Splice, Mis, O	

DNMT3A	2	2p23	yes		AML			L	Rec	Mis, F, N, S	
DUX4	4	4q35	yes		soft tissue sarcoma			M	Dom	T	CIC
EBF1	5	5q34	yes		lipoma			M	Dom	T	HMGA2
ECT2L	6	6q24.1	yes		ETP ALL			L	Rec	N, Splice, Mis	
EGFR	7	7p12.3-p12.1	yes	yes	glioma, NSCLC	NSCLC	familial lung cancer	E, O	Dom	A, O, Mis	
EIF4A2	3	3q27.3	yes		NHL			L	Dom	T	BCL6
ELF4	X	Xq26	yes		AML			L	Dom	T	ERG
ELK4	1	1q32	yes		prostate			E	Dom	T	SLC45A3
ELKS	12	12p13.3	yes		papillary thyroid			E	Dom	T	RET
ELL	19	19p13.1	yes		AL			L	Dom	T	MLL
ELN	7	7q11.23	yes		B-ALL			L	Dom	T	PAX5
EML4	2	2p21	yes		NSCLC			E	Dom	T	ALK
EP300	22	22q13	yes		colorectal, breast, pancreatic, AML, ALL, DLBCL			L, E	Rec	T, N, F, Mis, O	MLL, RUNXBP2
EPS15	1	1p32	yes		ALL			L	Dom	T	MLL
ERBB2	17	17q21.1	yes		breast, ovarian, other tumor types, NSCLC, gastric			E	Dom	A, Mis, O	

ERCC2	19	19q13.2-q13.3		yes		skin basal cell, skin squamous cell, melanoma	xeroderma pigmentosum (D)	E	Rec	Mis, N, F, S	
ERCC3	2	2q21		yes		skin basal cell, skin squamous cell, melanoma	xeroderma pigmentosum (B)	E	Rec	Mis, S	
ERCC4	16	16p13.3-p13.13		yes		skin basal cell, skin squamous cell, melanoma	xeroderma pigmentosum (F)	E	Rec	Mis, N, F	
ERCC5	13	13q33		yes		skin basal cell, skin squamous cell, melanoma	xeroderma pigmentosum (G)	E	Rec	Mis, N, F	
ERG	21	21q22.3	yes		Ewing sarcoma, prostate, AML			M, E, L	Dom	T	EWSR1, TMPRSS2, ELF4, FUS, HERPUD1, NDRG1
ETV1	7	7p22	yes		Ewing sarcoma, prostate			M, E	Dom	T	EWSR1, TMPRSS2, SLC45A3, C15orf21, HNRNPA2B1. ACSL3
ETV4	17	17q21	yes		Ewing sarcoma, prostate carcinoma			M, E	Dom	T	EWSR1, TMPRSS2, DDX5, KLK2, CANT1
ETV5	3	3q28	yes		prostate			E	Dom	T	TMPRSS2, SCL45A3

ETV6	12	12p13	yes		congenital fibrosarcoma, multiple leukemia and lymphoma, secretory breast, MDS, ALL			L, E, M	Dom	T	NTRK3, RUNX1, PDGFRB, ABL1, MN1, ABL2, FACL6, CHIC2, ARNT, JAK2, EVI1, CDX2, STL, HLXB9, MDS2, PER1, SYK, TTL, FGFR3, PAX5
EVI1	3	3q26	yes		AML, CML			L	Dom	T	RUNX1, ETV6, PRDM16, RPN1
EWSR1	22	22q12	yes		Ewing sarcoma, desmoplastic small round cell tumor , ALL, clear cell sarcoma, sarcoma, myoepithelioma			L, M	Dom	T	FLI1, ERG, ZNF278, NR4A3, FEV, ATF1, ETV1, ETV4, WT1, ZNF384, CREB1, POU5F1, PBX1
EXT1	8	8q24.11-q24.13		yes		exostoses, osteosarcoma	multiple exostoses type 1	M	Rec	Mis, N, F, S	
EXT2	11	11p12-p11		yes		exostoses, osteosarcoma	multiple exostoses type 2	M	Rec	Mis, N, F, S	
EZH2	7	7q35-q36	yes		DLBCL			L	Rec?	Mis	

EZR	6	6q25.3	yes		NSCLC			E	Dom	T	ROS1
FACL6	5	5q31	yes		AML, AEL			L	Dom	T	ETV6
FAM22A	10	10q23.2	yes		endometrial stromal sarcoma			M	Dom	T	YWHAE
FAM22B	10	10q22.3	yes		endometrial stromal sarcoma			M	Dom	T	YWHAE
FAM46C	1	1p12	yes		MM			L	Rec	Mis, F, O	
FANCA	16	16q24.3		yes		AML, leukemia	Fanconi anaemia A	L	Rec	D, Mis, N, F, S	
FANCC	9	9q22.3		yes		AML, leukemia	Fanconi anaemia C	L	Rec	D, Mis, N, F, S	
FANCD2	3	3p26		yes		AML, leukemia	Fanconi anaemia D2	L	Rec	D, Mis, N, F	
FANCE	6	6p21-p22		yes		AML, leukemia	Fanconi anaemia E	L	Rec	N, F, S	
FANCF	11	11p15		yes		AML, leukemia	Fanconi anaemia F	L	Rec	N, F	
FANCG	9	9p13		yes		AML, leukemia	Fanconi anaemia G	L	Rec	Mis, N, F, S	
FBXO11	2	2p16.3	yes		DLBCL			L	Rec	Mis, F, D	
FBXW7	4	4q31.3	yes		colorectal, endometrial, T-ALL			E, L	Rec	Mis, N, D, F	
FCGR2B	1	1q23	yes		ALL			L	Dom	T	?
FEV	2	2q36	yes		Ewing sarcoma			M	Dom	T	EWSR1, FUS

FGFR1	8	8p11.2-p11.1	yes		MPD, NHL			L	Dom	T	BCR, FOP, ZNF198, CEP1
FGFR1OP	6	6q27	yes		MPD, NHL			L	Dom	T	FGFR1
FGFR2	10	10q26	yes		gastric, NSCLC, endometrial			E	Dom	Mis	
FGFR3	4	4p16.3	yes		bladder, MM, T-cell lymphoma			L, E	Dom	Mis, T	IGH@, ETV6
FH	1	1q42.1		yes		leiomyomatosis, renal	hereditary leiomyomatosis and renal cell cancer	E, M	Rec	Mis, N, F	
FHIT	3	3p14.2	yes		pleomorphic salivary gland adenoma			E	Dom	T	HMGA2
FIP1L1	4	4q12	yes		idiopathic hypereosinophilic syndrome			L	Dom	T	PDGFRA
FLI1	11	11q24	yes		Ewing sarcoma			M	Dom	T	EWSR1
FLJ27352	15	15q21.3	yes		PMBL, Hodgkin lymphoma			L	Dom	T	CIITA
FLT3	13	13q12	yes		AML, ALL			L	Dom	Mis, O	
FNBP1	9	9q23	yes		AML			L	Dom	T	MLL
FOXL2	3	3q23	yes		granulosa-cell tumor of the ovary			O	Dom	Mis	

FOXO1A	13	13q14.1	yes		alveolar rhabdomyosarcoma			M	Dom	T	PAX3
FOXO3A	6	6q21	yes		AL			L	Dom	T	MLL
FOXP1	3	3p14.1	yes		ALL			L	Dom	T	PAX5
FSTL3	19	19p13	yes		B-CLL			L	Dom	T	CCND1
FUBP1	1	1p13.1	yes		oligodendroglioma			O	Rec	F, N	
FUS	16	16p11.2	yes		liposarcoma, AML, Ewing sarcoma, angiomatoid fibrous histiocytoma, fibromyxoid sarcoma			M, L	Dom	T	DDIT3, ERG, FEV, ATF1, CREB3L2, CREB3L1
FVT1	18	18q21.3	yes		B-NHL			L	Dom	T	IGK@
GAS7	17	17p	yes		AML*			L	Dom	T	MLL
GATA1	X	Xp11.23	yes		megakaryoblastic leukemia of Downs syndrome			L	Dom	Mis, F	
GATA2	3	3q21.3	yes		AML (CML blast transformation)			L	Dom	Mis	
GATA3	10	10p15	yes		breast			E	Rec	F, N, S	
GMPS	3	3q24	yes		AML			L	Dom	T	MLL
GNA11	19	19p13.3	yes		uveal melanoma			E	Dom	Mis	
GNAQ	9	9q21	yes		uveal			E	Dom	Mis	

					melanoma						
GNAS	20	20q13.2	yes		pituitary adenoma			E	Dom	Mis	
GOLGA5	14	14q	yes		papillary thyroid			E	Dom	T	RET
GOPC	6	6q21	yes		glioblastoma			O	Dom	O	ROS1
GPC3	X	Xq26.1		yes		Wilms tumor	Simpson-Golabi-Behmel syndrome	O	Rec/X	T, D, Mis, N, F, S	
GPHN	14	14q24	yes		AL			L	Dom	T	MLL
GRAF	5	5q31	yes		AML, MDS			L	Dom	T, F, S	MLL
H3F3A	1	1q42.12	yes		glioma			O	Dom	Mis	
HCMOGT-1	17	17p11.2	yes		JMML			L	Dom	T	PDGFRB
HEAB	11	11q12	yes		AML			L	Dom	T	MLL
HERPUD1	16	16q12.2-q13	yes		prostate			E	Dom	T	ERG
HEY1	8	8q21	yes		mesenchymal chondrosarcoma			M	Dom	T	NCOA2
HIP1	7	7q11.23	yes		CMML			L	Dom	T	PDGFRB
HIST1H3B	6	6p22.1	yes		glioma			O	Dom	Mis	
HIST1H4I	6	6p21.3	yes		NHL			L	Dom	T	BCL6
HLF	17	17q22	yes		ALL			L	Dom	T	TCF3
HLXB9	7	7q36	yes		AML			L	Dom	T	ETV6

HMGA1	6	6p21	yes		microfollicular thyroid adenoma, various benign mesenchymal tumors			E, M	Dom	T	?
HMGA2	12	12q15	yes		lipoma, leiomyoma, pleomorphic salivary gland adenoma			M	Dom	T	LHFP, RAD51L1, LPP, COX6C, CMKOR1, NFIB, ALDH2, CCNB1IP1, EBF1, WIF1, FHIT
HNRNPA2B1	7	7p15	yes		prostate			E	Dom	T	ETV1
HOOK3	8	8p11.21	yes		papillary thyroid			E	Dom	T	RET
HOXA11	7	7p15-p14.2	yes		CML			L	Dom	T	NUP98
HOXA13	7	7p15-p14.2	yes		AML			L	Dom	T	NUP98
HOXA9	7	7p15-p14.2	yes		AML*			L	Dom	T	NUP98, MSI2
HOXC11	12	12q13.3	yes		AML			L	Dom	T	NUP98
HOXC13	12	12q13.3	yes		AML			L	Dom	T	NUP98
HOXD11	2	2q31-q32	yes		AML			L	Dom	T	NUP98
HOXD13	2	2q31-q32	yes		AML*			L	Dom	T	NUP98

HRAS	11	11p15.5	yes	yes	infrequent sarcomas, rare other tumor types	rhabdomyosarcoma, ganglioneuroblastoma, bladder	Costello syndrome	E, L, M	Dom	Mis	
HRPT2	1	1q21-q31	yes	yes	parathyroid adenoma	parathyroid adenoma, multiple ossifying jaw fibroma	hyperparathyroidism-jaw tumor syndrome	E, M	Rec	Mis, N, F	
HSPCA	14	14q32.31	yes		NHL			L	Dom	T	BCL6
HSPCB	6	6p12	yes		NHL			L	Dom	T	BCL6
IDH1	2	2q33.3	yes		glioblastoma			O	Dom	Mis	
IDH2	15	15q26.1	yes		glioblastoma			M	Dom	M	
IGH@	14	14q32.33	yes		MM, Burkitt lymphoma, NHL, CLL, B-ALL, MALT, MLCLS			L	Dom	T	MYC, FGFR3,PAX5, IRTA1, IRF4, CCND1, BCL9, BCL8, BCL6, BCL2, BCL3, BCL10, BCL11A. LHX4, DDX6, NFKB2, PAFAH1B2, PCSK7, CRLF2
IGK@	2	2p12	yes		Burkitt lymphoma, B-NHL			L	Dom	T	MYC, FVT1

IGL@	22	22q11.1-q11.2	yes		Burkitt lymphoma			L	Dom	T	BCL9, MYC, CCND2
IKZF1	7	7p12.2	yes		ALL, DLBCL			L	Rec,Dom	D,T	BCL6
IL2	4	4q26-q27	yes		intestinal T-cell lymphoma			L	Dom	T	TNFRSF17
IL21R	16	16p11	yes		NHL			L	Dom	T	BCL6
IL6ST	5	5q11	yes		hepatocellular carcinoma			E	Dom	O	
IL7R	5	5p13	yes		ALL, ETP ALL			L	Dom	Mis, O	
IRF4	6	6p25-p23	yes		MM			L	Dom	T	IGH@
IRTA1	1	1q21	yes		B-NHL			L	Dom	T	IGH@
ITK	5	5q31-q32	yes		peripheral T-cell lymphoma			L	Dom	T	SYK
JAK1	1	1p32.3-p31.3	yes		ALL			L	Dom	Mis	
JAK2	9	9p24	yes		ALL, AML, MPD, CML			L	Dom	T, Mis, O	ETV6, PCM1, BCR
JAK3	19	19p13.1	yes		acute megakaryocytic leukemia, ETP ALL			L	Dom	Mis	
JAZF1	7	7p15.2-p15.1	yes		endometrial stromal tumor			M	Dom	T	SUZ12
JUN	1	1p32-p31	yes		sarcoma			M	Dom	A	

KCNJ5	11	11q24	yes	yes	adrenal	adrenal	Familial hyperaldosteronism type III	E		Mis	
KDM5A	12	12p11	yes		AML			L	Dom	T	NUP98
KDM5C	X	Xp11.22-p11.21	yes		clear cell renal carcinoma			E	Rec	N, F, S	
KDM6A	X	Xp11.2	yes		renal, oesophageal SCC, MM			E, L	Rec	D, N, F, S	
KDR	4	4q11-q12	yes		NSCLC, angiosarcoma			E	Dom	Mis	
KIAA1549	7	7q34	yes		pilocytic astrocytoma			O	Dom	O	BRAF
KIF5B	10	10p11.22	yes		NSCLC			E	Dom	T	RET, ALK
KIT	4	4q12	yes	yes	GIST, AML, TGCT, mastocytosis, mucosal melanoma	GIST, epithelioma	familial gastrointestinal stromal tumor	L, M, O, E	Dom	Mis, O	
KLF4	9	9q31	yes		meningioma			O		Mis	
KLK2	19	19q13.41	yes		prostate			E	Dom	T	ETV4
KRAS	12	12p12.1	yes		pancreatic, colorectal, lung, thyroid, AML,			L, E, M, O	Dom	Mis	

					other tumor types						
KTN1	14	14q22.1	yes		papillary thyroid			E	Dom	T	RET
LAF4	2	2q11.2-q12	yes		ALL, T-ALL			L	Dom	T	MLL, RUNX1
LASP1	17	17q11-q21.3	yes		AML			L	Dom	T	MLL
LCK	1	1p35-p34.3	yes		T-ALL			L	Dom	T	TRB@
LCP1	13	13q14.1-q14.3	yes		NHL			L	Dom	T	BCL6
LCX	10	10q21	yes		AML			L	Dom	T	MLL
LHFP	13	13q12	yes		lipoma			M	Dom	T	HMGA2
LIFR	5	5p13-p12	yes		salivary adenoma			E	Dom	T	PLAG1
LMO1	11	11p15	yes	yes	T-ALL, neuroblastoma	neuroblastoma		L	Dom	T, A	TRD@
LMO2	11	11p13	yes		T-ALL			L	Dom	T	TRD@
LPP	3	3q28	yes		lipoma, leukemia			L, M	Dom	T	HMGA2, MLL, C12orf9
LRIG3	12	12q14.1	yes		NSCLC			E	Dom	T	ROS1
LYL1	19	19p13.2-p13.1	yes		T-ALL			L	Dom	T	TRB@
MADH4	18	18q21.1	yes	yes	colorectal, pancreatic, small intestine	gastrointestinal polyp	juvenile polyposis	E	Rec	D, Mis, N, F	

MAF	16	16q22-q23	yes		MM			L	Dom	T	IGH@
MAFB	20	20q11.2-q13.1	yes		MM			L	Dom	T	IGH@
MALT1	18	18q21	yes		MALT			L	Dom	T	BIRC3
MAML2	11	11q22-q23	yes		salivary gland mucoepidermoid			E	Dom	T	MECT1, CRTC3
MAP2K1	15	15q22.1-q22.33	yes		NSCLC, melanoma, colorectal			E	Dom	Mis	
MAP2K2	19	19p13.3	yes		NSCLC, melanoma			E	Dom	Mis	
MAP2K4	17	17p11.2	yes		pancreatic, breast, colorectal			E	Rec	D, Mis, N	
MAX	14	14q23	yes	yes	pheochromocytoma	pheochromocytoma		E			
MDM2	12	12q15	yes		sarcoma, glioma, colorectal, other tumor types			M, O, E, L	Dom	A	
MDM4	1	1q32	yes		glioblastoma, bladder, retinoblastoma			M	Dom	A	
MDS1	3	3q26	yes		MDS, AML			L	Dom	T	RUNX1
MDS2	1	1p36	yes		MDS			L	Dom	T	ETV6

MECT1	19	19p13	yes		salivary gland mucoepidermoid			E	Dom	T	MAML2
MED12	X	Xq13	yes		uterine leiomyoma			M	Dom	M, S	
MEN1	11	11q13	yes	yes	parathyroid tumors, pancreatic neuroendocrine tumor	parathyroid adenoma, pituitary adenoma, pancreatic islet cell, carcinoid	multiple endocrine neoplasia type 1	E	Rec	D, Mis, N, F, S	
MET	7	7q31	yes		papillary renal, head-neck squamous cell	papillary renal	familial papillary renal cancer	E	Dom	Mis	
MITF	3	3p14.1	yes		melanoma			E	Dom	A	
MKL1	22	22q13	yes		acute megakaryocytic leukemia			L	Dom	T	RBM15
MLF1	3	3q25.1	yes		AML			L	Dom	T	NPM1
MLH1	3	3p21.3	yes	yes	colorectal, endometrial, ovarian, CNS	colorectal, endometrial, ovarian, CNS	hereditary non-polyposis colorectal cancer, Turcot syndrome	E, O	Rec	D, Mis, N, F, S	

MLL	11	11q23	yes		AML, ALL			L	Dom	T, O	MLL, MLLT1, MLLT2, MLLT3, MLLT4, MLLT7, MLLT10, MLLT6, ELL, EPS15, AF1Q, CREBBP, SH3GL1, FNBP1, PNUTL1, MSF, GPHN, GMPS, SSH3BP1, ARHGEF12, GAS7, FOXO3A, LAF4, LCX, SEPT6, LPP, CBFA2T1, GRAF, EP300, PICALM, HEAB
MLL2	12	12q12 -q14	yes		medulloblastoma, renal			O, E	Rec	N, F, Mis	
MLL3	7	7q36.1	yes		medulloblastoma			O	Rec	N	
MLLT1	19	19p13.3	yes		AL			L	Dom	T	MLL
MLLT10	10	10p12	yes		AL			L	Dom	T	MLL, PICALM, CDK6
MLLT2	4	4q21	yes		AL			L	Dom	T	MLL
MLLT3	9	9p22	yes		ALL			L	Dom	T	MLL

MLLT4	6	6q27	yes		AL			L	Dom	T	MLL
MLLT6	17	17q21	yes		AL			L	Dom	T	MLL
MLLT7	X	Xq13.1	yes		AL			L	Dom	T	MLL
MN1	22	22q13	yes		AML, meningioma			L, O	Dom	T	ETV6
MPL	1	p34	yes	yes	MPD	MPD	familial essential thrombocythemia	L	Dom	Mis	
MSF	17	17q25	yes		AML*			L	Dom	T	MLL
MSH2	2	2p22-p21	yes	yes	colorectal, endometrial, ovarian	colorectal, endometrial, ovarian	hereditary non-polyposis colorectal cancer	E	Rec	D, Mis, N, F, S	
MSH6	2	2p16	yes	yes	colorectal	colorectal, endometrial, ovarian	hereditary non-polyposis colorectal cancer	E	Rec	Mis, N, F, S	
MSI2	17	17q23.2	yes		CML			L	Dom	T	HOXA9
MSN	X	Xq11.2-q12	yes		ALCL			L	Dom	T	ALK
MTCP1	X	Xq28	yes		T cell prolymphocytic leukemia			L	Dom	T	TRA@
MUC1	1	1q21	yes		B-NHL			L	Dom	T	IGH@
MUTYH	1	1p34.3-1p32.1		yes		colorectal	adenomatous polyposis coli	E	Rec	Mis	

MYB	6	6q22-23	yes		adenoid cystic carcinoma			E	Dom	T	NFIB
MYC	8	8q24.12-q24.13	yes		Burkitt lymphoma, amplified in other cancers, B-CLL			L, E	Dom	A, T	IGK@, BCL5, BCL7A , BTG1, TRA@, IGH@
MYCL1	1	1p34.3	yes		small cell lung carcinoma			E	Dom	A	
MYCN	2	2p24.1	yes		neuroblastoma			O	Dom	A	
MYD88	3	3p22	yes		ABC-DLBCL			L	Dom	Mis	
MYH11	16	16p13.13-p13.12	yes		AML			L	Dom	T	CBFB
MYH9	22	22q13.1	yes		ALCL			L	Dom	T	ALK
MYST4	10	10q22	yes		AML			L	Dom	T	CREBBP
NACA	12	12q23-q24.1	yes		NHL			L	Dom	T	BCL6
NBS1	8	8q21		yes		NHL, glioma, medulloblastoma, rhabdomyosarcoma	Nijmegen breakage syndrome	L, E, M, O	Rec	Mis, N, F	
NCOA1	2	2p23	yes		alveolar rhabdomyosarcoma			M	Dom	T	PAX3
NCOA2	8	8q13.1	yes		AML, chondrosarcoma			L	Dom	T	RUNXBP2, HEY1

NCOA4	10	10q11.2	yes		papillary thyroid			E	Dom	T	RET
NDRG1	8	8q24.3	yes		prostate			E	Dom	T	ERG
NF1	17	17q12	yes	yes	neurofibroma, glioma	neurofibroma, glioma	neurofibromatosis type 1	O	Rec	D, Mis, N, F, S, O	
NF2	22	22q12.2	yes	yes	meningioma, acoustic neuroma, renal	meningioma, acoustic neuroma	neurofibromatosis type 2	O	Rec	D, Mis, N, F, S, O	
NFE2L2	2	2q31	yes		NSCLC, HNSCC			E	Dom	Mis	
NFIB	9	9p24.1	yes		adenoid cystic carcinoma, lipoma			E	Dom	T	MYB, HGMA2
NFKB2	10	10q24	yes		B-NHL			L	Dom	T	IGH@
NIN	14	14q24	yes		MPD			L	Dom	T	PDGFRB
NKX2-1	14	14q13	yes		NSCLC			E	Dom	A	
NONO	X	Xq13.1	yes		papillary renal			E	Dom	T	TFE3
NOTCH1	9	9q34.3	yes		T-ALL			L	Dom	T, Mis, O	TRB@
NOTCH2	1	1p13-p11	yes		marginal zone lymphoma, DLBCL			L	Dom	N, F, Mis	
NPM1	5	5q35	yes		NHL, APL, AML			L	Dom	T, F	ALK, RARA, MLF1
NR4A3	9	9q22	yes		extraskeletal myxoid chondrosarcoma			M	Dom	T	EWSR1

NRAS	1	1p13.2	yes		melanoma, MM, AML, thyroid			L, E	Dom	Mis	
NSD1	5	5q35	yes		AML			L	Dom	T	NUP98
NT5C2	10	10q24.32	yes		relapse ALL			L		Mis	
NTRK1	1	1q21-q22	yes		papillary thyroid			E	Dom	T	TPM3, TPR, TFG
NTRK3	15	15q25	yes		congenital fibrosarcoma, secretory breast			E, M	Dom	T	ETV6
NUMA1	11	11q13	yes		APL			L	Dom	T	RARA
NUP214	9	9q34.1	yes		AML, T-ALL			L	Dom	T	DEK, SET, ABL1
NUP98	11	11p15	yes		AML			L	Dom	T	HOXA9, NSD1, WHSC1L1, DDX10, TOP1, HOXD13, PMX1, HOXA13, HOXD11, HOXA11, RAP1GDS1, HOXC11
OLIG2	21	21q22.11	yes		T-ALL			L	Dom	T	TRA@
OMD	9	9q22.31	yes		aneurysmal bone cyst			M	Dom	T	USP6
P2RY8	X,Y	Xp22.3; Yp11.3	yes		B-ALL, Down syndrome associated			L	Dom	T	CRLF2

					ALL						
PAFAH1 B2	11	11q23	yes		MLCLS			L	Dom	T	IGH@
PALB2	16	16p12 .1		yes		Wilms tumor, medullobl astoma, AML ,breast	Fanconi anaemia N, breast cancer susceptibil ity	L, O, E	Rec	F, N, Mis	
PAX3	2	2q35	yes		alveolar rhabdomy osarcoma			M	Dom	T	FOXO1A, NCOA1
PAX5	9	9p13	yes		NHL, ALL, B-ALL			L	Dom	T, Mis, D, F, S	IGH@, ETV6, PML, FOXP1, ZNF521, ELN
PAX7	1	1p36. 2- p36.1 2	yes		alveolar rhabdomy osarcoma			M	Dom	T	FOXO1A
PAX8	2	2q12- q14	yes		follicular thyroid			E	Dom	T	PPARG
PBRM1	3	3p21	yes		clear cell renal carcinoma , breast			E	Rec	Mis, N, F, S, D, O	
PBX1	1	1q23	yes		pre B-ALL, myoepithe lioma			L, M	Dom	T	TCF3, EWSR1
PCM1	8	8p22- p21.3	yes		papillary thyroid, CML, MPD			E, L	Dom	T	RET, JAK2
PCSK7	11	11q23 .3	yes		MLCLS			L	Dom	T	IGH@

PDE4DIP	1	1q12	yes		MPD			L	Dom	T	PDGFRB
PDGFB	22	22q12.3-q13.1	yes		DFSP			M	Dom	T	COL1A1
PDGFRA	4	4q11-q13	yes		GIST, idiopathic hypereosinophilic syndrome, paediatric glioblastoma			L, M, O	Dom	Mis, O, T	FIP1L1
PDGFRB	5	5q31-q32	yes		MPD, AML, CMML, CML			L	Dom	T	ETV6, TRIP11, HIP1, RAB5EP, H4, NIN, HCMOGT-1, PDE4DIP
PER1	17	17p13.1-17p12	yes		AML, CMML			L	Dom	T	ETV6
PHF6	X	Xq26.3	yes		ETP ALL			L	Rec	F, N, Splice, Mis	
PHOX2B	4	4p12	yes	yes	neuroblastoma	neuroblastoma	familial neuroblastoma	O	Rec	Mis, F	
PICALM	11	11q14	yes		TALL, AML,			L	Dom	T	MLLT10, MLL
PIK3CA	3	3q26.3	yes		colorectal, gastric, glioblastoma, breast			E, O	Dom	Mis	
PIK3R1	5	5q13.1	yes		glioblastoma, ovarian,			E, O	Rec	Mis, F, O	

					colorectal						
PIM1	6	6p21.2	yes		NHL			L	Dom	T	BCL6
PLAG1	8	8q12	yes		salivary adenoma			E	Dom	T	TCEA1, LIFR, CTNNB1, CHCHD7
PML	15	15q22	yes		APL, ALL			L	Dom	T	RARA, PAX5
PMS1	2	2q31-q33		yes		colorectal, endometrial, ovarian	hereditary non-polyposis colorectal cancer	E	Rec	Mis, N	
PMS2	7	7p22		yes		colorectal, endometrial, ovarian, medulloblastoma, glioma	hereditary non-polyposis colorectal cancer, Turcot syndrome	E	Rec	Mis, N, F	
PMX1	1	1q24	yes		AML			L	Dom	T	NUP98
PNUTL1	22	22q11.2	yes		AML			L	Dom	T	MLL
POT1	7	7q31.33	yes		CLL			L		Mis, N	
POU2AF1	11	11q23.1	yes		NHL			L	Dom	T	BCL6
POU5F1	6	6p21.31	yes		sarcoma			M	Dom	T	EWSR1
PPARG	3	3p25	yes		follicular thyroid			E	Dom	T	PAX8
PPP2R1A	19	19q13.41	yes		clear cell ovarian carcinoma			E	Dom?	Mis	
PRCC	1	1q21.	yes		papillary			E	Dom	T	TFE3

		1			renal						
PRDM1	6	6q21	yes		DLBCL			L	Rec	D, N, Mis, F, S	
PRDM16	1	1p36.23-p33	yes		MDS, AML			L	Dom	T	EVI1
PRF1	10	10q22		yes		various leukemia, lymphoma		L	Rec	M	
PRKAR1A	17	17q23-q24	yes	yes	papillary thyroid	myxoma, endocrine, papillary thyroid	Carney complex	E, M	Dom, Rec	T, Mis, N, F, S	RET
PRO1073	11	11q31.1	yes		renal cell carcinoma (childhood epithelioid)			E	Dom	T	TFEB
PSIP2	9	9p22.2	yes		AML			L	Dom	T	NUP98
PTCH	9	9q22.3	yes	yes	skin basal cell, medulloblastoma	skin basal cell, medulloblastoma	nevoid basal cell carcinoma syndrome	E, M	Rec	Mis, N, F, S	
PTEN	10	10q23.3	yes	yes	glioma, prostate, endometrial	harmartoma, glioma, prostate, endometrial	Cowden syndrome, Bannayan-Riley-Ruvalcaba syndrome	L, E, M, O	Rec	D, Mis, N, F, S	
PTPN11	12	12q24.1	yes		JMML, AML, MDS			L	Dom	Mis	
RAB5EP	17	17p13	yes		CMML			L	Dom	T	PDGFRB
RAC1	7	7p22	yes		melanoma			E		Mis	

RAD51L1	14	14q23-q24.2	yes		lipoma, uterine leiomyoma			M	Dom	T	HMGA2
RAF1	3	3p25	yes		pilocytic astrocytoma			M	Dom	T	SRGAP3
RALGDS	9	9q34.3	yes		PMBL, Hodgkin lymphoma,			L	Dom	T	CIITA
RANBP17	5	5q34	yes		ALL			L	Dom	T	TRD@
RAP1GDS1	4	4q21-q25	yes		T-ALL			L	Dom	T	NUP98
RARA	17	17q12	yes		APL			L	Dom	T	PML, ZNF145, TIF1, NUMA1, NPM1
RB1	13	13q14	yes	yes	retinoblastoma, sarcoma, breast, small cell lung carcinoma	retinoblastoma, sarcoma, breast, small cell lung carcinoma	familial retinoblastoma	L, E, M, O	Rec	D, Mis, N, F, S	
RBM15	1	1p13	yes		acute megakaryocytic leukemia			L	Dom	T	MKL1
RECQL4	8	8q24.3		yes		osteosarcoma, skin basal cell, skin sqamous cell	Rothmund-Thompson syndrome	M	Rec	N, F, S	

REL	2	2p13-p12	yes		Hodgkin lymphoma			L	Dom	A	
RET	10	10q11.2	yes	yes	medullary thyroid, papillary thyroid, pheochromocytoma, NSCLC	medullary thyroid, papillary thyroid, pheochromocytoma	multiple endocrine neoplasia 2A/2B	E, O	Dom	T, Mis, N, F	H4, PRKAR1A, NCOA4, PCM1, GOLGA5, TRIM33, KTN1, TRIM27, HOOK3, KIF5B, CCDC6
RNF43	17	17q22	yes		cholangiocarcinoma, ovary, pancreas			E	Rec	Mis, N	
ROS1	6	6q22	yes		glioblastoma, NSCLC			O, E	Dom	T	GOPC, SDC4, SLC34A2, EZR, LRIG3
RPL10	X	Xq28	yes		T-ALL			L		Mis	
RPL22	1	1p36.31	yes		AML, CML			L	Dom	T	RUNX1
RPL5	1	1p22.1	yes		T-ALL			L		Mis, N, F	
RPN1	3	3q21.3-q25.2	yes		AML			L	Dom	T	EVI1
RUNDC2A	16	16p13.13	yes		PMBL, Hodgkin lymphoma			L	Dom	T	CIITA
RUNX1	21	21q22.3	yes		AML, preB- ALL, T-ALL			L	Dom	T	RPL22, MDS1, EVI1, CBFA2T3, CBFA2T1, ETV6, LAF4

RUNXBP2	8	8p11	yes		AML			L	Dom	T	CREBBP, NCOA2, EP300
SBDS	7	7q11		yes		AML, MDS	Schwachman-Diamond syndrome	L	Rec	Gene Conversion	
SDC4	20	20q12	yes		NSCLC			E	Dom	T	ROS1
SDH5	11	11q12.2		yes		paraganglioma	familial paraganglioma	M	Rec	M	
SDHB	1	1p36.1-p35		yes		paraganglioma, pheochromocytoma	familial paraganglioma	O	Rec	Mis, N, F	
SDHC	1	1q21		yes		paraganglioma, pheochromocytoma	familial paraganglioma	O	Rec	Mis, N, F	
SDHD	11	11q23		yes		paraganglioma, pheochromocytoma	familial paraganglioma	O	Rec	Mis, N, F, S	
SEPT6	X	Xq24	yes		AML			L	Dom	T	MLL
SET	9	9q34	yes		AML			L	Dom	T	NUP214
SETBP1	18	18q21.1	yes		atypical CML			L		Mis	NUP98
SETD2	3	3p21.31	yes		clear cell renal carcinoma			E	Rec	N, F, S, Mis	
SF3B1	2	2q33.1	yes		myelodysplastic syndrome			L	Dom	Mis	
SFPQ	1	1p34.3	yes		papillary renal			E	Dom	T	TFE3
SFRS3	6	6p21	yes		follicular lymphoma			L	Dom	T	BCL6

SH2B3	12	12q24.12	yes		MPD, sAML, erythrocytosis, B-ALL			L	Rec?	Mis, F,N	
SH3GL1	19	19p13.3	yes		AL			L	Dom	T	MLL
SIL	1	1p32	yes		T-ALL			L	Dom	T	TAL1
SLC34A2	4	4p15.2	yes		NSCLC			E	Dom	T	ROS1
SLC45A3	1	1q32	yes		prostate			E	Dom	T	ETV1, ETV5, ELK4, ERG
SMARCA4	19	19p13.2	yes		NSCLC			E	Rec	F, N, Mis	
SMARCB1	22	22q11	yes	yes	malignant rhabdoid	malignant rhabdoid	rhabdoid predisposition syndrome	M	Rec	D, N, F, S	
SMARCE1	17	17q21.2		yes		meningioma		O			
SMO	7	7q31-q32	yes		skin basal cell			E	Dom	Mis	
SOCS1	16	16p13.13	yes		Hodgkin lymphoma, PMBL			L	Rec	F, O	
SOX2	3	3q26.3-q27	yes		NSCLC, oesophageal squamous carcinoma			E	Dom	A	
SRGAP3	3	3p25.3	yes		pilocytic astrocytoma			M	Dom	T	RAF1
SRSF2	17	17q25	yes		MDS, CLL			L	Dom	Mis	
SS18	18	18q11.2	yes		synovial sarcoma			M	Dom	T	SSX1, SSX2

SS18L1	20	20q13.3	yes		synovial sarcoma			M	Dom	T	SSX1
SSH3BP1	10	10p11.2	yes		AML			L	Dom	T	MLL
SSX1	X	Xp11.23-p11.22	yes		synovial sarcoma			M	Dom	T	SS18
SSX2	X	Xp11.23-p11.22	yes		synovial sarcoma			M	Dom	T	SS18
SSX4	X	Xp11.23	yes		synovial sarcoma			M	Dom	T	SS18
STAT3	17	17q21.31	yes		T-cell large granular lymphocytic lymphoma			L	Dom	Mis,O	
STK11	19	19p13.3	yes	yes	NSCLC, pancreatic	jejunal hamartoma, ovarian, testicular, pancreatic	Peutz-Jeghers syndrome	E, M, O	Rec	D, Mis, N, F, S	
STL	6	6q23	yes		B-ALL			L	Dom	T	ETV6
SUFU	10	10q24.32	yes	yes	medulloblastoma	medulloblastoma	medulloblastoma predisposition	O	Rec	D, F, S	
SUZ12	17	17q11.2	yes		endometrial stromal tumor			M	Dom	T	JAZF1
SYK	9	9q22	yes		MDS, peripheral T-cell lymphoma			L	Dom	T	ETV6, ITK

TAF15	17	17q11.1-q11.2	yes		extraskeletal myxoid chondrosarcoma, ALL			L, M	Dom	T	TEC, CHN1, ZNF384
TAL1	1	1p32	yes		lymphoblastic leukemia/ biphasic			L	Dom	T	TRD@, SIL
TAL2	9	9q31	yes		T-ALL			L	Dom	T	TRB@
TCEA1	8	8q11.2	yes		salivary adenoma			E	Dom	T	PLAG1
TCF1	12	12q24.2	yes	yes	hepatic adenoma, hepatocellular	hepatic adenoma, hepatocellular carcinoma	familial hepatic adenoma	E	Rec	Mis, F	
TCF12	15	15q21	yes		extraskeletal myxoid chondrosarcoma			M	Dom	T	TEC
TCF3	19	19p13.3	yes		pre B-ALL			L	Dom	T	PBX1, HLF, TFPT
TCF7L2	10	10q25.3	yes		colorectal			E	Dom	T	VTI1A
TCL1A	14	14q32.1	yes		T-CLL			L	Dom	T	TRA@
TCL6	14	14q32.1	yes		T-ALL			L	Dom	T	TRA@
TERT	5	5p15.33	yes	yes	melanoma	melanoma		E			
TET2	4	4q24	yes		MDS			L	Rec	Mis N, F	
TFE3	X	Xp11.22	yes		papillary renal, alveolar soft part sarcoma,			E	Dom	T	SFPQ, ASPSCR1, PRCC, NONO, CLTC

					renal						
TFEB	6	6p21	yes		renal cell carcinoma (childhood epithelioid)			E,M	Dom	T	ALPHA
TFG	3	3q11-q12	yes		papillary thyroid, ALCL, NSCLC			E, L	Dom	T	NTRK1, ALK
TFPT	19	19q13	yes		pre-B ALL			L	Dom	T	TCF3
TFRC	3	3q29	yes		NHL			L	Dom	T	BCL6
THRAP3	1	1p34.3	yes		aneurysmal bone cyst			M	Dom	T	USP6
TIF1	7	7q32-q34	yes		APL			L	Dom	T	RARA
TLX1	10	10q24	yes		T-ALL			L	Dom	T	TRB@, TRD@
TLX3	5	5q35.1	yes		T-ALL			L	Dom	T	BCL11B
TMPRSS2	21	21q22.3	yes		prostate			E	Dom	T	ERG, ETV1, ETV4, ETV5
TNFAIP3	6	6q23	yes		marginal zone B-cell lymphomas, Hodgkin lymphoma, PMBL			L	Rec	D, N, F	
TNFRSF14	1	1p36.32	yes		follicular lymphoma			L	Rec	Mis, N, F	
TNFRSF17	16	16p13.1	yes		intestinal T-cell lymphoma			L	Dom	T	IL2

TNFRSF 6	10	10q24 .1	yes		TGCT, nasal NK/T lymphoma , skin squamous cell carcinoma -burn scar related			L, E, O	Rec	Mis	
TOP1	20	20q12 -q13.1	yes		AML*			L	Dom	T	NUP98
TP53	17	17p13	yes	yes	breast, colorectal, lung, sarcoma, adrenocort ical, glioma, multiple other tumor types	breast, sarcoma, adrenocort ical carcinoma , glioma, multiple other tumor types	Li-Fraumeni syndrome	L, E, M, O	Rec	Mis, N, F	
TPM3	1	1q22-q23	yes		papillary thyroid, ALCL, NSCLC			E, L	Dom	T	NTRK1, ALK, ROS1
TPM4	19	19p13 .1	yes		ALCL			L	Dom	T	ALK
TPR	1	1q25	yes		papillary thyroid			E	Dom	T	NTRK1
TRA@	14	14q11 .2	yes		T-ALL			L	Dom	T	ATL,OLIG2, MYC, TCL1A, TCL6, MTCP1, TCL6

TRAF7	16	16p13.3	yes		meningioma			O		Mis	
TRB@	7	7q35	yes		T-ALL			L	Dom	T	HOX11, LCK, NOTCH1, TAL2, LYL1
TRD@	14	14q11	yes		T-cell leukemia			L	Dom	T	TAL1, HOX11, TLX1, LMO1, LMO2, RANBP17
TRIM27	6	6p22	yes		papillary thyroid			E	Dom	T	RET
TRIM33	1	1p13	yes		papillary thyroid			E	Dom	T	RET
TRIP11	14	14q31-q32	yes		AML			L	Dom	T	PDGFRB
TSC1	9	9q34	yes	yes	renal cell carcinoma, bladder carcinoma	hamartoma, renal cell carcinoma, tuberous sclerosis tuber	Tuberous sclerosis 1	E, O	Rec	D, Mis, N, F, S	
TSC2	16	16p13.3	yes	yes	pulmonary lymphangioleiomyomatosis (LAM), renal angiomyolipoma and head and neck cancer	hamartoma, renal cell carcinoma, tuberous sclerosis tuber	Tuberous sclerosis 2	E, O	Rec	D, Mis, N, F, S	
TSHR	14	14q31	yes	yes	toxic thyroid adenoma	thyroid adenoma		E	Dom	Mis	
TTL	2	2q13	yes		ALL			L	Dom	T	ETV6

U2AF1	21	21q22.3	yes		CLL, MDS			L	Dom	Mis	
USP6	17	17p13	yes		aneurysmal bone cyst			M	Dom	T	COL1A1, CDH11, ZNF9, OMD, THRAP3
VHL	3	3p25	yes	yes	renal, haemangioma, pheochromocytoma	renal, haemangioma, pheochromocytoma	Von Hippel-Lindau syndrome	E, M, O	Rec	D, Mis, N, F, S	
VTI1A	10	10q25.2	yes		colorectal			E	Dom	T	TCF7L2
WAS	X	Xp11.23-p11.22				lymphoma	Wiskott-Aldrich syndrome	L	X-linked recessive	Mis, N, F, S	
WHSC1	4	4p16.3	yes		MM			L	Dom	T	IGH@
WHSC1L1	8	8p12	yes		AML			L	Dom	T	NUP98
WIF1	12	12q14.3	yes		pleomorphic salivary gland adenoma			E	Dom	T	HMGA2
WRN	8	8p12-p11.2		yes		osteosarcoma, meningioma, other tumor types	Werner syndrome	L, E, M, O	Rec	Mis, N, F, S	

WT1	11	11p13	yes	yes	Wilms tumor, desmoplastic small round cell tumor	Wilms tumor	Denys-Drash syndrome, Frasier syndrome, familial Wilms tumor	O	Rec	D, Mis, N, F, S	EWSR1
WTX	X	Xq11.1	yes		Wilms tumor			O	Rec	F, D, N, Mis	
WWTR1	3	3q23-q24	yes		epithelioid haemangioendothelioma			M	Dom	T	CAMTA1
XPA	9	9q22.3		yes		skin basal cell, skin squamous cell, melanoma	xeroderma pigmentosum (A)	E	Rec	Mis, N, F, S	
XPC	3	3p25		yes		skin basal cell, skin squamous cell, melanoma	xeroderma pigmentosum (C)	E	Rec	Mis, N, F, S	
XPO1	2	2p15	yes		CLL			L	Dom	Mis	
YWHAE	17	17p13.3	yes		edometrial stromal sarcoma			M	Dom	T	FAM22a, FAM22B
ZNF145	11	11q23.1	yes		APL			L	Dom	T	RARA
ZNF198	13	13q11-q12	yes		MPD, NHL			L	Dom	T	FGFR1
ZNF278	22	22q12-q14	yes		Ewing sarcoma			M	Dom	T	EWSR1
ZNF331	19	19q13.3-q13.4	yes		follicular thyroid adenoma			E	Dom	T	?

ZNF384	12	12p13	yes		ALL			L	Dom	T	EWSR1, TAF15
ZNF521	18	18q11.2	yes		ALL			L	Dom	T	PAX5
ZNF9	3	3q21	yes		aneurysmal bone cyst			M	Dom	T	USP6
ZRSR2	X	Xp22.1	yes		MDS, CLL			L	Rec	F, S, Mis	

Abbreviation	Term
A	amplification
AEL	acute eosinophilic leukemia
AL	acute leukemia
ALCL	anaplastic large-cell lymphoma
ALL	acute lymphocytic leukemia
AML	acute myelogenous leukemia
AML*	acute myelogenous leukemia (primarily treatment associated)
sAML	secondary acute myelogenous leukemia
APL	acute promyelocytic leukemia
B-ALL	B-cell acute lymphocytic leukemia
B-CLL	B-cell Lymphocytic leukemia
B-NHL	B-cell non-Hodgkin lymphoma
CLL	chronic lymphatic leukemia
CML	chronic myeloid leukemia
CMML	chronic myelomonocytic leukemia
CNS	central nervous system
D	large deletion
DFSP	dermatofibrosarcoma protuberans
DLBCL	diffuse large B-cell lymphoma

DLCL	diffuse large-cell lymphoma
Dom	dominant
E	epithelial
F	frameshift
GIST	gastrointestinal stromal tumor
JMML	juvenile myelomonocytic leukemia
L	leukemia/lymphoma
M	mesenchymal
MALT	mucosa-associated lymphoid tissue lymphoma
MDS	myelodysplastic syndrome
Mis	missense
MLCLS	mediastinal large cell lymphoma with sclerosis
MM	multiple myeloma
MPD	myeloproliferative disorder
N	nonsense
NHL	non-Hodgkin lymphoma
NK/T	natural killer T cell
NSCLC	non small cell lung cancer
O	other
PMBL	primary mediastinal B-cell lymphoma
pre-B All	pre-B-cell acute lymphoblastic leukemia
RCC	renal cell carcinoma
Rec	recessive
S	splice site
T	translocation
T-ALL	T-cell acute lymphoblastic leukemia
T-CLL	T-cell chronic lymphocytic leukemia
TGCT	testicular germ cell tumor
T-PLL	T cell prolymphocytic leukemia

Suppl. Tab. 2. Manually curated list of genes not known to be associated with cancer (nCan).

nCan Genes
BAAT
BDNF
FGD1
GLUD1
HSPG2
PCDH15
AAAS
AANAT
ABCC2
ABCD3
ABHD5
ACAA1
ACF
ACSL6
AHI1
AIRE
ALDH3A2
ALMS1
ALOX5
AMH
AMHR2
ANCR
ANK2
ANO6
AP3B1
APBB2
ARL13B
ARX
ASCL1
ATP13A2

ATP7B
ATXN3
AVP
B3GALTL
B4GALT7
BAG3
BBS1
BBS10
BBS12
BBS2
BBS4
BBS5
BBS7
BBS9
BCS1L
BEAN1
BEST1
BLOC1S3
BSND
C1GALT1C1
CACNA1C
CACNB2
CC2D2A
CCBE1
CD46
CD96
CDH23
CFH
CHAT
CHD7
CHRND
CHRNE
CHST14

CHUK
CISD2
CLCF1
CLCNKA
CLCNKB
CLRN1
CNNM4
CNTNAP2
COL11A1
COL11A2
COL18A1
COL1A2
COL2A1
COL3A1
COL4A4
COL4A5
COL5A1
COL5A2
CRLF1
CTSC
DCAF17
DDX11
DGUOK
DHCR7
DHODH
DLX3
DNMT3B
EDN3
EHMT1
EIF2AK3
EPHX1
ERCC1
ERCC6

ERCC8
ESCO2
EVC
EVC2
EYA1
FAM20C
FAM58A
FBN1
FLNB
FLVCR2
FMR1
FOXC2
FOXE1
FOXG1
FRAS1
FREM2
FST
FTL
GDF6
GDNF
GLE1
GLI3
GP1BA
GP1BB
GP9
GPD1L
HCN4
HDAC4
HMX1
HPRT1
HPS1
HPS3
HPS4

HPS5
HPS6
HYLS1
IQCB1
IRF6
JAG1
KAL1
KCNA1
KCNE1
KCNE2
KCNE3
KCNH2
KCNJ1
KCNJ10
KCNJ2
KCNQ1
KIAA1279
LIG4
LMX1B
LOR
LPIN2
LRP2
LRP4
LRP5
LRPPRC
LYST
MAOA
MBTPS2
MECP2
MGP
MID1
MKKS
MKS1

MLPH
MPV17
MUSK
MYO5A
MYO7A
NDN
NDUFA2
NDUFS3
NDUFS4
NDUFS7
NDUFS8
NDUFV1
NHS
NIPBL
NLRP12
NLRP3
NPHS1
NPHS2
NR2E3
NSDHL
OBSL1
OCRL
OFD1
PER2
PEX1
PEX10
PEX13
PEX14
PEX16
PEX19
PEX2
PEX3
PIP5K1C

PITX2
PKP1
PLOD1
PLOD2
POLA1
POLR1C
POLR1D
POR
PQBP1
PRG4
PRKAG2
PROK2
PROKR2
PVRL1
PVRL4
RAB23
RAB27A
RAB3GAP1
RAB3GAP2
RAC2
RAI1
RAPSN
RBM10
RBM28
RELN
RFX5
RFXAP
RNASEH2A
RNASEH2B
RNASEH2C
RNF135
RNF168
RPGRIP1L

SALL1
SALL4
SCARB2
SCN3B
SCN4B
SCN5A
SCNN1B
SCNN1G
SH3PXD2B
SHANK3
SHOC2
SHROOM4
SIL1
SIX1
SIX5
SLC12A3
SLC16A2
SLC19A2
SLC25A15
SLC26A4
SLC27A4
SLC2A10
SLC39A13
SLC6A8
SMC1A
SMC3
SNAP29
SNRPN
SNTA1
SOX10
SOX18
SPG20
SPINK5

SPRED1
ST3GAL5
SUCLA2
SUCLG1
SURF1
TAP1
TAP2
TAPBP
TAR
TBCE
TBX1
TBX15
TBX3
TBX4
TBX5
TCF4
TCOF1
TFAP2A
TFAP2B
TH
TMEM216
TMEM67
TREX1
TRPS1
TSPYL1
TWIST1
TYMP
UBR1
UGT1A1
USH1C
USH1G
USH2A
VANGL1

VCAN
VPS13B
WDPCP
WFS1
WHCR
XK
ZEB2
ZNF469

Suppl. Tab. 3. List of all the human genes with citations >= 10 and OncoScore > 21.09.

Genes with citations >= 10 and OncoScore > 21.09 (5945)
A4GALT
AASS
AATF
AATK
ABCA13
ABCA2
ABCA5
ABCA6
ABCA8
ABCB1
ABCB10
ABCB5
ABCB6
ABCB8
ABCC1
ABCC10
ABCC11
ABCC12
ABCC2
ABCC3
ABCC4
ABCC5
ABCE1
ABCF1
ABCF2
ABCG2

ABHD11
ABHD2
ABI3BP
ABL1
ABL2
ABLIM1
ABTB1
ACAA1
ACADSB
ACAP1
ACCS
ACER2
ACIN1
ACKR3
ACLY
ACOX2
ACOXL
ACPP
ACRBP
ACRC
ACSL3
ACSL4
ACSL5
ACSM1
ACSS1
ACSS2
ACTA2
ACTB
ACTG2
ACTL6A

ACTL6B
ACTN1
ACTN4
ACTR1A
ACVR1
ACVR1B
ACVR1C
ACVR2A
ACVRL1
ACY1
ACYP2
ADAM10
ADAM11
ADAM12
ADAM15
ADAM17
ADAM19
ADAM21
ADAM23
ADAM28
ADAM29
ADAM7
ADAM8
ADAM9
ADAMDEC1
ADAMTS1
ADAMTS12
ADAMTS14
ADAMTS15
ADAMTS16

ADAMTS18
ADAMTS2
ADAMTS20
ADAMTS3
ADAMTS4
ADAMTS6
ADAMTS7
ADAMTS8
ADAMTS9
ADAMTSL3
ADARB2
ADC
ADCY2
ADD3
ADH1A
ADH1B
ADH1C
ADH7
ADHFE1
ADI1
ADIPOR1
ADIPOR2
ADNP2
ADRM1
AEBP1
AEBP2
AFAP1
AFAP1L2
AFF1
AFF3

AFF4
AFP
AGAP1
AGAP2
AGFG1
AGGF1
ago-02
AGPAT9
AGR2
AGR3
AGT
AHCY
AHCYL1
AHNAK
AHR
AHRR
AHSA1
AICDA
AIFM1
AIFM2
AIM1
AIM2
AIMP1
AIMP2
AIP
AJAP1
AJUBA
AK5
AKAP12
AKAP13

AKAP4
AKAP8
AKAP8L
AKAP9
AKIP1
AKNA
AKR1A1
AKR1B1
AKR1B10
AKR1C1
AKR1C2
AKR1C3
AKR1C4
AKR7A2
AKR7A3
AKT1
AKT1S1
AKT2
AKT3
AKTIP
ALAS1
ALCAM
ALDH1A1
ALDH1A3
ALDH1B1
ALDH1L1
ALDH2
ALDH3A1
ALDH3B1
ALDH6A1

ALDOA
ALDOB
ALK
ALKBH1
ALKBH2
ALKBH3
ALKBH8
ALOX12
ALOX15
ALOX15B
ALPP
ALX1
ALX4
ALYREF
AMACR
AMBRA1
AMER1
AMFR
AMH
AMHR2
AMOT
AMOTL1
AMOTL2
AMY2A
ANAPC1
ANAPC10
ANAPC11
ANAPC2
ANAPC4
ANAPC5

ANAPC7
ANGPT1
ANGPT2
ANGPT4
ANGPTL1
ANGPTL2
ANGPTL4
ANGPTL5
ANGPTL7
ANKHD1
ANKRD11
ANKRD17
ANKRD2
ANKRD26
ANKRD28
ANKRD6
ANKS1B
ANLN
ANO1
ANO10
ANO6
ANO7
ANP32A
ANP32B
ANP32E
ANTXR1
ANTXR2
ANXA1
ANXA10
ANXA11

ANXA2
ANXA3
ANXA4
ANXA5
ANXA6
ANXA7
ANXA8
AP1B1
AP2S1
AP3B2
APAF1
APBA2
APBA3
APBB1IP
APBB2
APC
APC2
APCS
APEH
APEX1
APEX2
API5
APIP
APITD1
APLF
APOA1BP
APOBEC1
APOBEC2
APOBEC3A
APOBEC3B

APOBEC3C
APOBEC3D
APOBEC3H
APOD
APRT
APTX
ARAP1
ARAP2
ARCN1
AREG
ARF6
ARFGAP3
ARFGEF1
ARHGAP1
ARHGAP12
ARHGAP15
ARHGAP18
ARHGAP20
ARHGAP21
ARHGAP22
ARHGAP24
ARHGAP26
ARHGAP29
ARHGAP31
ARHGAP35
ARHGAP4
ARHGAP5
ARHGAP8
ARHGAP9
ARHGDIA

ARHGDIB
ARHGEF1
ARHGEF10
ARHGEF11
ARHGEF12
ARHGEF17
ARHGEF18
ARHGEF2
ARHGEF3
ARHGEF4
ARHGEF5
ARHGEF7
ARID1A
ARID1B
ARID2
ARID3A
ARID3B
ARID4A
ARID4B
ARID5B
ARIH2
ARL11
ARL2
ARL2BP
ARL4C
ARL5B
ARL6IP1
ARL6IP5
ARMC5
ARMC8

ARNT
ARPC1B
ARRB1
ARRDC3
ARVCF
ASAH1
ASAP1
ASAP2
ASAP3
ASB2
ASB3
ASB4
ASB9
ASCL1
ASCL2
ASF1A
ASF1B
ASH1L
ASH2L
ASIP
ASNA1
ASNS
ASPG
ASPSCR1
ASRGL1
ASS1
ASTL
ASTN1
ASXL1
ASXL2

ASXL3
ATAD2
ATAD3A
ATAD3B
ATAD5
ATAT1
ATF1
ATF2
ATF3
ATF4
ATF5
ATF6
ATF7
ATF7IP
ATG101
ATG12
ATG13
ATG14
ATG16L2
ATG2B
ATG3
ATG4A
ATG4C
ATG4D
ATG5
ATG7
ATG9A
ATG9B
ATIC
ATM

ATMIN
ATOH1
ATP1B1
ATP1B2
ATP2A3
ATP2B3
ATP4A
ATP4B
ATP5A1
ATP5B
ATP5J
ATP5O
ATP6V0A1
ATP6V0B
ATP6V0C
ATP6V1C1
ATR
ATRIP
ATRX
AURKA
AURKB
AURKC
AXIN1
AXIN2
AXL
AZGP1
AZIN1
AZU1
B2M
B3GALT5

B3GNT1
B3GNT5
B3GNT8
B4GALNT2
B4GALT1
B4GALT5
BAALC
BABAM1
BACH1
BACH2
BAG3
BAG4
BAG5
BAG6
BAGE
BAI1
BAI2
BAI3
BAIAP3
BAK1
BAMBI
BANF1
BANP
BAP1
BARD1
BARX2
BATF
BATF2
BATF3
BAX

BAZ1A
BAZ1B
BAZ2A
BBC3
BBS9
BBX
BCAM
BCAN
BCAP31
BCAR1
BCAR3
BCAR4
BCAS1
BCAS2
BCAS3
BCAS4
BCAT1
BCCIP
BCL10
BCL11A
BCL11B
BCL2
BCL2A1
BCL2L1
BCL2L10
BCL2L11
BCL2L12
BCL2L13
BCL2L14
BCL2L15

BCL2L2
BCL3
BCL6
BCL6B
BCL7A
BCL7B
BCL9
BCL9L
BCLAF1
BCOR
BCORL1
BCR
BDH2
BDKRB1
BDP1
BECN1
BEND3
BET1L
BEX1
BEX2
BFAR
BHLHE40
BHLHE41
BID
BIK
BIN1
BIN3
BIRC2
BIRC3
BIRC5

BIRC6
BIRC7
BIRC8
BLCAP
BLID
BLM
BLMH
BLNK
BLOC1S2
BLVRB
BLZF1
BMF
BMI1
BMP10
BMP2K
BMP6
BMP8A
BMPR1A
BMPR1B
BMX
BNC1
BNC2
BNIP1
BNIP2
BNIP3
BNIP3L
BNIPL
BOD1
BOP1
BPIFA1

BPIFA2
BPTF
BRAF
BRAP
BRAT1
BRCA1
BRCA2
BRCC3
BRD1
BRD2
BRD3
BRD4
BRD7
BRD8
BRD9
BRDT
BRE
BRF1
BRF2
BRI3
BRIP1
BRK1
BRMS1
BRPF1
BRS3
BRSK1
BRSK2
BSG
BST2
BTBD1

BTBD9
BTC
BTF3
BTG1
BTG2
BTG3
BTG4
BTK
BTLA
BTN3A1
BTN3A3
BTRC
BUB1
BUB1B
BUB3
BUD31
BYSL
C10orf10
C11orf30
C11orf95
C12orf5
C14orf166
C19orf10
C19orf40
C1GALT1
C1GALT1C1
C1QTNF1
C1QTNF3
C1QTNF5
C1QTNF6

C22orf29
C2orf40
C5AR2
C8orf4
C9orf156
C9orf3
CA11
CA12
CA13
CA7
CA8
CA9
CAB39
CAB39L
CABIN1
CABLES1
CABYR
CACNA1G
CACNA2D2
CACNA2D3
CACUL1
CACYBP
CADM1
CADM2
CADM3
CADM4
CAGE1
CALCA
CALCOCO1
CALD1

CALM2
CALM3
CALML3
CALR
CALU
CAMK1D
CAMK2G
CAMK2N1
CAMKK1
CAMKK2
CAMLG
CAMTA1
CAND1
CANT1
CAPG
CAPN14
CAPN2
CAPN5
CAPN6
CAPN8
CAPN9
CAPNS1
CAPRIN1
CAPRIN2
CAPZA1
CAPZA2
CARD10
CARD11
CARD14
CARD6

CARD8
CARD9
CARM1
CASC1
CASC3
CASC5
CASP1
CASP10
CASP12
CASP14
CASP2
CASP3
CASP4
CASP5
CASP6
CASP7
CASP8
CASP8AP2
CASP9
CASS4
CASZ1
CAV1
CBFA2T2
CBFA2T3
CBFB
CBL
CBLB
CBLL1
CBLN4
CBR1

CBR3
CBX1
CBX2
CBX3
CBX4
CBX5
CBX6
CBX7
CBX8
CBY1
CC2D1A
CCAR1
CCAR2
CCBE1
CCDC50
CCDC6
CCDC8
CCDC80
CCDC88A
CCHCR1
CCIN
CCKBR
CCL1
CCL13
CCL14
CCL15
CCL16
CCL17
CCL18
CCL19

CCL2
CCL20
CCL21
CCL22
CCL23
CCL25
CCL27
CCL3
CCL5
CCL7
CCL8
CCM2
CCNA1
CCNA2
CCNB1
CCNB1IP1
CCNB2
CCNB3
CCNC
CCND1
CCND2
CCND3
CCNDBP1
CCNE1
CCNE2
CCNF
CCNG1
CCNG2
CCNH
CCNK

CCNL1
CCNL2
CCNO
CCNT2
CCP110
CCR1
CCR10
CCR2
CCR4
CCR6
CCR7
CCR8
CCR9
CCRL2
CCT2
CCT3
CCT4
CCT5
CCT6A
CD109
CD14
CD151
CD160
CD163
CD164
CD177
CD180
CD19
CD1A
CD1C

CD1D
CD2
CD200
CD200R1
CD207
CD209
CD22
CD226
CD24
CD244
CD247
CD248
CD27
CD274
CD276
CD28
CD300A
CD300C
CD320
CD33
CD34
CD37
CD38
CD3D
CD3E
CD3EAP
CD3G
CD4
CD40
CD40LG

CD44
CD46
CD47
CD48
CD5
CD52
CD53
CD55
CD58
CD59
CD5L
CD6
CD63
CD68
CD69
CD7
CD70
CD72
CD74
CD79A
CD79B
CD80
CD81
CD82
CD83
CD84
CD86
CD8A
CD9
CD93

CD96
CD97
CD99
CDA
CDC123
CDC14A
CDC14B
CDC16
CDC20
CDC23
CDC25A
CDC25B
CDC25C
CDC27
CDC34
CDC37
CDC42
CDC42BPA
CDC42BPB
CDC42EP3
CDC42EP4
CDC42EP5
CDC45
CDC5L
CDC6
CDC7
CDC73
CDCA2
CDCA3
CDCA5

CDCA7
CDCA7L
CDCA8
CDCP1
CDH1
CDH10
CDH11
CDH13
CDH15
CDH16
CDH17
CDH18
CDH19
CDH2
CDH3
CDH4
CDH5
CDH6
CDK1
CDK10
CDK11A
CDK12
CDK13
CDK14
CDK16
CDK19
CDK2
CDK2AP1
CDK2AP2
CDK3

CDK4
CDK5R2
CDK5RAP1
CDK5RAP3
CDK6
CDK7
CDK8
CDK9
CDKL2
CDKN1A
CDKN1B
CDKN1C
CDKN2A
CDKN2AIP
CDKN2B
CDKN2C
CDKN2D
CDKN3
CDO1
CDON
CDR2
CDS1
CDT1
CDX1
CDX2
CDX4
CDYL
CEACAM1
CEACAM3
CEACAM5

CEACAM6
CEACAM7
CEACAM8
CEBPA
CEBPB
CEBPD
CEBPE
CEBPG
CELF1
CELF2
CENPA
CENPE
CENPF
CENPH
CENPI
CENPJ
CENPK
CENPT
CENPW
CEP131
CEP135
CEP152
CEP164
CEP170
CEP192
CEP250
CEP290
CEP350
CEP55
CEP57

CEP63
CEP97
CERK
CERS1
CERS2
CERS3
CERS4
CERS5
CERS6
CES2
CETN1
CETN2
CFC1
CFL1
CFLAR
CGA
CGB
CGB5
CGGBP1
CHAC1
CHAF1A
CHAF1B
CHCHD4
CHD1
CHD1L
CHD4
CHD5
CHD6
CHD8
CHD9

CHEK1
CHEK2
CHERP
CHFR
CHGA
CHGB
CHI3L1
CHI3L2
CHIC2
CHKA
CHM
CHMP1A
CHMP2A
CHMP3
CHMP4C
CHP2
CHRDL2
CHRNA3
CHRNA5
CHRNB4
CHST1
CHST10
CHST11
CHST15
CHST2
CHST3
CHST7
CHTF18
CHTOP
CHUK

CIAO1
CIAPIN1
CIB1
CIDEB
CIDEC
CIITA
CIRBP
CISD2
CISH
CITED1
CITED2
CITED4
CIZ1
CKAP2
CKAP4
CKAP5
CKB
CKLF
CKS1B
CKS2
CLASP2
CLCA2
CLCA4
CLDN1
CLDN10
CLDN12
CLDN18
CLDN2
CLDN23
CLDN3

CLDN4
CLDN5
CLDN6
CLDN7
CLDN8
CLDN9
CLEC11A
CLEC12A
CLEC1B
CLEC2B
CLEC2D
CLEC4C
CLEC5A
CLEC9A
CLIC1
CLIC3
CLIC4
CLIC5
CLIC6
CLIP1
CLIP4
CLK1
CLK2
CLK3
CLLU1
CLMP
CLNK
CLPTM1
CLPTM1L
CLSPN

CLSTN1
CLTA
CLTB
CLTC
CLTCL1
CLU
CMBL
CMKLR1
CMTM3
CMTM5
CMTM7
CMTM8
CNDP2
CNKSR1
CNKSR2
CNKSR3
CNN1
CNN2
CNN3
CNOT1
CNOT2
CNOT3
CNOT6
CNOT6L
CNOT7
CNOT8
CNRIP1
CNST
CNTF
CNTFR

CNTN1
CNTRL
CNTROB
COBLL1
COL11A1
COL11A2
COL12A1
COL16A1
COL18A1
COL21A1
COL23A1
COL4A2
COL4A3BP
COL4A6
COMMD1
COPS2
COPS3
COPS5
COPS6
CORO1C
COX4I1
COX6C
COX7A1
COX7B
COX7C
CPA3
CPA4
CPEB2
CPEB4
CPO

CPSF6
CPT1C
CR1
CR2
CRABP1
CRABP2
CRADD
CRB2
CRB3
CRBN
CREB1
CREB3
CREB3L1
CREB3L2
CREB3L4
CREB5
CREBBP
CREBL2
CREBZF
CRELD2
CRIP1
CRIP2
CRISP3
CRK
CRKL
CRLF2
CRMP1
CRNN
CRTAM
CRTC1

CRTC2
CRTC3
CSAG2
CSDE1
CSE1L
CSF1
CSF1R
CSF2
CSF2RB
CSF3
CSF3R
CSGALNACT1
CSH2
CSK
CSMD1
CSMD2
CSMD3
CSNK1A1
CSNK1E
CSNK2A1
CSNK2B
CSPG4
CSPP1
CSRP1
CSRP2
CST1
CST6
CST7
CSTA
CT45A1

CTAG2
CTAGE1
CTAGE5
CTBP1
CTBP2
CTC1
CTCF
CTCFL
CTDSP1
CTDSP2
CTDSPL
CTF1
CTGF
CTHRC1
CTLA4
CTNNA1
CTNNA3
CTNNAL1
CTNNB1
CTNNBIP1
CTNND1
CTNND2
CTPS1
CTRB1
CTRC
CTSB
CTSD
CTSE
CTSL
CTSO

CTSZ
CTTN
CTTNBP2
CUBN
CUEDC2
CUL1
CUL2
CUL3
CUL4A
CUL4B
CUL5
CUL7
CUX1
CUZD1
CX3CL1
CXCL1
CXCL10
CXCL11
CXCL12
CXCL13
CXCL14
CXCL16
CXCL17
CXCL2
CXCL3
CXCL5
CXCL6
CXCL9
CXCR1
CXCR2

CXCR3
CXCR4
CXCR5
CXCR6
CXXC1
CXXC4
CXXC5
CYB561
CYB5B
CYCS
CYFIP2
CYGB
CYLD
CYP17A1
CYP19A1
CYP1A1
CYP1B1
CYP24A1
CYP26A1
CYP27B1
CYP2A13
CYP2A6
CYP2A7
CYP2E1
CYP2F1
CYP2R1
CYP2S1
CYP2W1
CYP39A1
CYP3A43

CYP3A7
CYP4B1
CYP4F11
CYP4F3
CYP4X1
CYP4Z1
CYR61
CYTL1
D2HGDH
DAAM1
DAAM2
DAB2
DAB2IP
DACH1
DACH2
DACT1
DACT2
DACT3
DAND5
DAP3
DAPK1
DAPK2
DAPK3
DAXX
DAZAP2
DBF4
DBN1
DCAF6
DCAF7
DCBLD2

DCC
DCHS1
DCK
DCLK1
DCLRE1A
DCLRE1B
DCLRE1C
DCTD
DCUN1D1
DCXR
DDB1
DDB2
DDIT3
DDIT4
DDIT4L
DDR1
DDR2
DDX1
DDX10
DDX11
DDX17
DDX20
DDX21
DDX39A
DDX3X
DDX41
DDX42
DDX43
DDX46
DDX5

DDX53
DDX58
DDX6
DDX60
DEAF1
DEC1
DEDD
DEDD2
DEF6
DEFA4
DEFA5
DEFA6
DEFB126
DEK
DENND1A
DENND1B
DENND4A
DENR
DEPDC1
DEPDC5
DEPTOR
DERL1
DERL2
DFFA
DFFB
DFNA5
DGCR8
DHCR24
DHFR
DHRS2

DHRS3
DHRS4
DHRS9
DHX15
DHX32
DHX36
DHX9
DIABLO
DIAPH1
DIAPH2
DIAPH3
DICER1
DIDO1
DIRAS3
DIS3
DIS3L2
DIXDC1
DKC1
DKK1
DKK2
DKK3
DKK4
DLC1
DLD
DLEC1
DLEU7
DLG1
DLG2
DLG3
DLG5

DLGAP5
DLK1
DLL1
DLL4
DLX4
DMAP1
DMBT1
DMKN
DMTF1
DNAJA1
DNAJA3
DNAJA4
DNAJB1
DNAJB11
DNAJB4
DNAJB6
DNAJB9
DNAJC1
DNAJC10
DNAJC15
DNAJC2
DNAJC7
DNASE1L3
DND1
DNER
DNM1L
DNM3
DNMT1
DNMT3A
DNMT3B

DNMT3L
DOCK1
DOCK10
DOCK11
DOCK2
DOCK3
DOCK4
DOCK5
DOCK8
DOCK9
DOHH
DOK1
DOK2
DOK3
DOK4
DONSON
DOT1L
DPAGT1
DPCR1
DPEP1
DPF2
DPF3
DPH1
DPH2
DPP9
DPPA2
DPPA4
DPPA5
DPY30
DPYD

DPYS
DPYSL3
DPYSL5
DRAM1
DRAM2
DRAXIN
DRC1
DRG1
DROSHA
DSC2
DSC3
DSG2
DSN1
DTX1
DTX3L
DUOX1
DUOX2
DUSP1
DUSP10
DUSP12
DUSP14
DUSP16
DUSP2
DUSP22
DUSP23
DUSP26
DUSP27
DUSP3
DUSP4
DUSP5

DUSP6
DUSP7
DUSP8
DUSP9
DUX4
DVL1
DVL2
DVL3
DYNC1LI1
DYNLL2
DYNLRB1
DYNLRB2
DYRK1A
DYRK1B
DYRK2
DYRK3
DZIP1
E2F1
E2F2
E2F3
E2F4
E2F5
E2F6
E2F7
E2F8
E4F1
EAF1
EAF2
EBAG9
EBF1

EBF3
EBI3
EBNA1BP2
EBP
ECH1
ECHS1
ECM1
ECSCR
ECT2
EDA2R
EDAR
EDARADD
EDC4
EDEM3
EDIL3
EDNRB
EEA1
EED
EEF1A1
EEF1A2
EEF1D
EEF1E1
EEF2K
EFEMP1
EFEMP2
EFHD1
EFNA1
EFNA3
EFNA4
EFNA5

EFNB1
EFNB2
EFNB3
EFS
EGF
EGFL6
EGFL7
EGFR
EGLN1
EGLN2
EGLN3
EGR1
EGR3
EGR4
EHBP1
EHD1
EHD2
EHD3
EHD4
EHMT1
EHMT2
EI24
EID1
EIF1AX
EIF2A
EIF2AK2
EIF2AK3
EIF2AK4
EIF2S1
EIF2S3

EIF3A
EIF3D
EIF3E
EIF3F
EIF3H
EIF3I
EIF3M
EIF4A1
EIF4A2
EIF4A3
EIF4B
EIF4E
EIF4E2
EIF4E3
EIF4EBP1
EIF4EBP2
EIF4EBP3
EIF4ENIF1
EIF4G1
EIF4G2
EIF5A
EIF5A2
EIF6
ELAC1
ELAC2
ELANE
ELAVL1
ELAVL2
ELAVL3
ELAVL4

ELF1
ELF2
ELF3
ELF4
ELF5
ELK1
ELK3
ELK4
ELL
ELL2
ELP2
ELP3
ELTD1
EMCN
EME1
EMILIN1
EMILIN2
EMILIN3
EML1
EML2
EML4
EMP1
EMP2
EMP3
EN2
ENAH
ENAM
ENC1
ENDOG
ENDOU

ENDOV
ENO1
ENOSF1
ENOX1
ENOX2
ENPEP
ENPP2
ENTPD1
ENTPD5
ENY2
EOMES
EP300
EP400
EPAS1
EPB41L3
EPB41L4B
EPC1
EPC2
EPCAM
EPGN
EPHA1
EPHA10
EPHA2
EPHA3
EPHA6
EPHA7
EPHA8
EPHB1
EPHB2
EPHB3

EPHB4
EPHB6
EPM2AIP1
EPO
EPOR
EPS15
EPS8
EPSTI1
EPT1
ERAP1
ERAP2
ERBB2
ERBB2IP
ERBB3
ERBB4
ERC1
ERCC1
ERCC2
ERCC3
ERCC4
ERCC5
ERCC6
ERCC6L
ERCC8
EREG
ERGIC2
ERI3
ERLIN2
ERN1
ERN2

ERP29
ERRFI1
ESCO1
ESD
ESM1
ESPL1
ESR1
ESR2
ESRP1
ESRP2
ESRRA
ESRRB
ESRRG
ETS1
ETS2
ETV1
ETV3
ETV4
ETV5
ETV6
ETV7
EVI2A
EVI2B
EVI5
EVPL
EVX1
EWSR1
EXO1
EXOC2
EXOC3

EXOC4
EXOC5
EXOC8
EXOSC5
EXT1
EXT2
EXTL1
EXTL2
EXTL3
EYA2
EYA4
EYS
EZH1
EZH2
EZR
F10
F11R
F2R
F2RL1
F2RL3
FABP1
FABP5
FABP7
FADD
FAF1
FAIM2
FAIM3
FAM107A
FAM129A
FAM132A

FAM134B
FAM13A
FAM162A
FAM175A
FAM3B
FAM3C
FAM46A
FAM46C
FAM57A
FAM83A
FAM84B
FAM96B
FAN1
FANCA
FANCB
FANCC
FANCD2
FANCE
FANCF
FANCG
FANCI
FANCL
FANCM
FAP
FARP2
FAS
FASLG
FASN
FASTK
FAT1

FAT2
FAT3
FAT4
FBL
FBLIM1
FBLN1
FBLN2
FBN3
FBXL2
FBXL5
FBXL7
FBXO11
FBXO18
FBXO3
FBXO31
FBXO4
FBXO45
FBXO5
FBXO6
FBXW11
FBXW7
FBXW8
FCER1G
FCGBP
FCGR1A
FCGR2A
FCGR3A
FCGR3B
FCRL1
FCRL2

FCRL4
FCRL5
FCRLA
FDCSP
FDFT1
FDXR
FECH
FEM1A
FEM1B
FEN1
FERMT1
FERMT2
FERMT3
FETUB
FEZ1
FGD5
FGF1
FGF11
FGF12
FGF13
FGF19
FGF2
FGF20
FGF3
FGF4
FGF5
FGF6
FGF7
FGFBP1
FGFR1

FGFR1OP
FGFR1OP2
FGFR2
FGFR3
FGFR4
FGL1
FGL2
FHIT
FHL2
FHL3
FHOD1
FHOD3
FIGF
FILIP1
FILIP1L
FIP1L1
FIZ1
FJX1
FKBP11
FKBP3
FKBP4
FKBP8
FKBPL
FLCN
FLI1
FLII
FLNA
FLNC
FLOT1
FLOT2

FLT1
FLT3
FLT4
FLVCR1
FLVCR2
FMN1
FMN2
FMNL1
FMNL2
FMNL3
FN1
FNBP1
FNBP1L
FNBP4
FNDC1
FNDC3B
FNIP1
FNIP2
FNTA
FNTB
FOLH1
FOLR1
FOLR2
FOLR3
FOS
FOSL1
FOSL2
FOXA1
FOXA2
FOXA3

FOXC1
FOXC2
FOXD1
FOXD2
FOXD4
FOXE1
FOXF1
FOXF2
FOXH1
FOXJ2
FOXK1
FOXK2
FOXL1
FOXL2
FOXM1
FOXN1
FOXN2
FOXN3
FOXO1
FOXO3
FOXO4
FOXP1
FOXP3
FOXP4
FOXQ1
FOXR1
FOXR2
FPGS
FPR2
FPR3

FRAT1
FRAT2
FRK
FRMD3
FRMD4A
FRMD6
FRS2
FRS3
FRZB
FSCN1
FSTL1
FTH1
FTMT
FUBP1
FUCA1
FURIN
FUS
FUT1
FUT3
FUT4
FUT6
FUT7
FUT8
FXR1
FXYD3
FXYD5
FXYD6
FYCO1
FYN
FZD1

FZD10
FZD2
FZD3
FZD4
FZD5
FZD6
FZD7
FZD8
FZD9
FZR1
G0S2
G3BP1
G3BP2
GAB1
GAB2
GAB3
GABARAPL1
GABARAPL2
GABPA
GABRP
GADD45A
GADD45B
GADD45G
GADD45GIP1
GAGE1
GAGE7
GAL3ST2
GALM
GALNT1
GALNT10

GALNT12
GALNT14
GALNT3
GALNT7
GAN
GANC
GAPDH
GART
GAS1
GAS2
GAS6
GAS7
GAS8
GATA1
GATA2
GATA3
GATA5
GATA6
GATAD2B
GBP1
GBP5
GCFC2
GCLC
GCLM
GCNT1
GCNT2
GCNT3
GCSH
GDF10
GDF15

GDF2
GDF3
GDI2
GEM
GEMIN2
GEMIN4
GEMIN6
GEMIN7
gen-01
GFAP
GFER
GFI1
GFI1B
GFRA1
GFRA4
GGCT
GGH
GGN
GGNBP2
GGT1
GHDC
GINS1
GINS2
GINS3
GIPC1
GIPC2
GIPC3
GJC1
GKN1
GKN2

GLCE
GLG1
GLI1
GLI2
GLI3
GLIPR1
GLIPR2
GLIS1
GLIS2
GLMN
GLRX3
GLS2
GLTSCR2
GLUL
GMEB1
GMEB2
GMIP
GMNN
GMPR
GNA11
GNA12
GNA13
GNAI2
GNAO1
GNAQ
GNAS
GNB2
GNB2L1
GNG11
GNG12

GNG2
GNG7
GNL3
GNL3L
GNLY
GNMT
GOLGA2
GOLGA5
GOLM1
GOLPH3
GORASP1
GOT1
GP5
GP6
GPA33
GPC1
GPC3
GPC5
GPC6
GPER1
GPI
GPM6B
GPN1
GPNMB
GPR101
GPR110
GPR124
GPR125
GPR15
GPR160

GPR31
GPR34
GPR4
GPR55
GPR56
GPR64
GPR65
GPR68
GPR78
GPR87
GPRC5A
GPRC5B
GPRC5C
GPRC5D
GPS2
GPSM2
GPX1
GPX2
GPX3
GPX4
GPX6
GPX7
GRAMD4
GRAP2
GRB10
GRB14
GRB2
GRB7
GREB1
GREM1

GREM2
GRHL1
GRHL2
GRHL3
GRID2IP
GRINA
GRIP1
GRM1
GRP
GRPR
GSAP
GSC
GSDMA
GSDMB
GSG2
GSK3A
GSK3B
GSPT1
GSTA1
GSTA2
GSTA4
GSTA5
GSTM1
GSTM2
GSTM3
GSTM4
GSTM5
GSTO1
GSTO2
GSTP1

GSTZ1
GTF2H1
GTF2H5
GTSE1
GTSF1
GUCA2B
GUCY1A3
GUCY2C
GYLTL1B
GZMA
GZMB
GZMH
GZMK
GZMM
H2AFX
H2AFY
H2AFY2
H2AFZ
H3F3B
HABP4
HACE1
HAPLN1
HAS1
HAS2
HAS3
HAVCR2
HAX1
HBEGF
HBP1
HBZ

HCCS
HCFC1
HCK
HCLS1
HCST
HDAC1
HDAC10
HDAC11
HDAC2
HDAC3
HDAC4
HDAC5
HDAC6
HDAC7
HDAC8
HDAC9
HDC
HDGF
HDLBP
HEBP1
HECA
HECTD1
HEG1
HELLS
HELQ
HELT
HEMGN
HEPACAM
HERC1
HERC2

HERC3
HERC4
HERC5
HERPUD1
HES1
HES2
HES3
HES4
HES5
HES6
HEXIM1
HEY1
HEY2
HFM1
HGD
HGF
HHAT
HHEX
HHIP
HIC1
HIC2
HIF1A
HIF1AN
HIF3A
HINT1
HINT2
HIP1
HIPK1
HIPK2
HIPK3

HIVEP1
HIVEP2
HJURP
HK1
HK2
HK3
HLF
HLTF
HMBOX1
HMG20B
HMGA1
HMGA2
HMGB1
HMGB2
HMGB3
HMGCS1
HMGN1
HMGN2
HMGN3
HMGN5
HMMR
HMOX1
HN1
HNF1A
HNF1B
HNF4A
HNF4G
HNRNPA1
HNRNPA2B1
HNRNPA3

HNRNPC
HNRNPD
HNRNPF
HNRNPH1
HNRNPH2
HNRNPK
HNRNPL
HNRNPM
HNRNPU
HNRNPUL1
HOOK2
HOOK3
HOPX
HORMAD1
HORMAD2
HOXA1
HOXA10
HOXA11
HOXA13
HOXA4
HOXA5
HOXA6
HOXA7
HOXA9
HOXB13
HOXB2
HOXB3
HOXB4
HOXB5
HOXB6

HOXB7
HOXB8
HOXB9
HOXC10
HOXC11
HOXC12
HOXC13
HOXC4
HOXC5
HOXC6
HOXC8
HOXC9
HOXD1
HOXD10
HOXD13
HOXD3
HOXD4
HOXD8
HOXD9
HPCA
HPCAL1
HPD
HPGD
HPR
HPRT1
HPSE
HPSE2
HRAS
HRASLS
HRG

HRH2
HRK
HRNR
HRSP12
HS3ST1
HS3ST2
HS6ST2
HSBP1
HSD17B1
HSD17B12
HSD17B2
HSD17B6
HSD17B7
HSD3B1
HSD3B2
HSF1
HSF2
HSP90AA1
HSP90AB1
HSP90B1
HSPA12A
HSPA1A
HSPA1B
HSPA1L
HSPA2
HSPA4
HSPA4L
HSPA5
HSPA6
HSPA7

HSPA8
HSPA9
HSPB1
HSPB11
HSPB2
HSPB8
HSPBP1
HSPD1
HSPE1
HSPH1
HTATIP2
HTR3C
HTRA2
HTRA3
HUNK
HUS1
HUS1B
HUWE1
HVCN1
HYAL1
HYAL2
HYAL3
HYAL4
HYOU1
IARS2
IBTK
ICAM1
ICAM2
ICAM3
ICMT

ICOS
ICOSLG
ID1
ID2
ID3
ID4
IDH1
IDH2
IDO1
IDO2
IER2
IER3
IER5
IFI16
IFI27
IFI30
IFI35
IFI44
IFI6
IFIT1
IFIT2
IFIT3
IFIT5
IFITM1
IFITM2
IFITM3
IFNA1
IFNA10
IFNA17
IFNA2

IFNA8
IFNAR1
IFNAR2
IFNB1
IFNG
IFNGR1
IFNGR2
IFNK
IFNLR1
IFRD1
IFT88
IGBP1
IGF1
IGF1R
IGF2
IGF2BP1
IGF2BP3
IGF2R
IGFBP1
IGFBP2
IGFBP3
IGFBP4
IGFBP5
IGFBP6
IGFBP7
IGHG1
IGJ
IGLL1
IGSF11
IGSF3

IGSF8
IKBKB
IKBKE
IKBKG
IKZF1
IKZF2
IKZF3
IL10
IL10RA
IL10RB
IL11
IL11RA
IL12A
IL13
IL13RA1
IL13RA2
IL15
IL15RA
IL16
IL17A
IL17B
IL17C
IL17F
IL17RA
IL17RB
IL17RC
IL17RD
IL18
IL18R1
IL19

IL1A
IL1B
IL1F10
IL1R1
IL1R2
IL1RAP
IL1RN
IL2
IL20
IL21
IL21R
IL22RA1
IL22RA2
IL23A
IL24
IL26
IL27
IL27RA
IL2RA
IL2RB
IL2RG
IL3
IL31
IL31RA
IL32
IL36G
IL3RA
IL4
IL4I1
IL4R

IL5
IL5RA
IL6
IL6R
IL6ST
IL7
IL7R
IL8
IL9
IL9R
ILF2
ILF3
ILK
ILKAP
IMP3
IMPDH2
INCA1
INCENP
ING1
ING2
ING3
ING4
ING5
INHBA
INHBB
INHBC
INHBE
INO80
INPP4B
INPP5A

INPP5D
INPPL1
INSM1
INSR
INTS3
INTS6
IP6K2
IPMK
IPO13
IPO7
IPO8
IQGAP1
IQGAP2
IQGAP3
IQSEC1
IRAK1
IRAK2
IRAK3
IRAK4
IREB2
IRF1
IRF2
IRF2BP2
IRF3
IRF4
IRF7
IRF8
IRF9
IRG1
IRS1

IRS2
IRS4
IRX2
ISG15
ISG20
ITGA1
ITGA10
ITGA11
ITGA2
ITGA3
ITGA4
ITGA5
ITGA6
ITGA7
ITGA9
ITGAE
ITGAL
ITGAM
ITGAV
ITGAX
ITGB1
ITGB1BP1
ITGB2
ITGB3
ITGB3BP
ITGB4
ITGB5
ITGB6
ITGB7
ITGB8

ITIH2
ITIH3
ITIH4
ITIH5
ITK
ITPKA
ITPKB
ITPR2
ITSN2
IVL
IVNS1ABP
IWS1
JAG1
JAG2
JAK1
JAK2
JAK3
JAM2
JAM3
JARID2
JAZF1
JDP2
JMJD1C
JMJD6
JMY
JTB
JUNB
JUND
JUP
KANK1

KAT2A
KAT2B
KAT5
KAT6A
KAT6B
KAT7
KAT8
KCNH1
KCNH4
KCNH5
KCNIP3
KCNIP4
KCNJ5
KCNK12
KCNRG
KCP
KCTD10
KCTD11
KCTD13
KDM1A
KDM2A
KDM2B
KDM3A
KDM3B
KDM4A
KDM4B
KDM4C
KDM4D
KDM5A
KDM5B

KDM5C
KDM6A
KDM6B
KDM7A
KDM8
KDR
KEAP1
KHDC3L
KHDRBS1
KHDRBS2
KHDRBS3
KHSRP
KIAA0020
KIAA0101
KIAA1524
KIAA1549
KIDINS220
KIF11
KIF14
KIF15
KIF18A
KIF1B
KIF20A
KIF20B
KIF22
KIF23
KIF24
KIF27
KIF2A
KIF2B

KIF2C
KIF3C
KIF4A
KIF5B
KIFAP3
KIFC1
KIR2DL1
KIR2DL2
KIR2DL3
KIR2DL4
KIR2DS2
KIR3DL1
KIR3DL2
KIR3DS1
KISS1
KISS1R
KIT
KITLG
KLB
KLF1
KLF10
KLF11
KLF12
KLF16
KLF17
KLF2
KLF3
KLF4
KLF5
KLF6

KLF8
KLF9
KLHL20
KLHL6
KLK1
KLK10
KLK11
KLK12
KLK13
KLK14
KLK15
KLK2
KLK3
KLK4
KLK5
KLK6
KLK7
KLK8
KLK9
KLKB1
KLLN
KLRB1
KLRC1
KLRC2
KLRC3
KLRD1
KLRF1
KLRG1
KLRK1
KMT2A

KMT2B
KMT2C
KMT2D
KNTC1
KPNA2
KPNA4
KPNA6
KPNB1
KRAS
KREMEN1
KREMEN2
KRIT1
KRT1
KRT13
KRT14
KRT15
KRT16
KRT17
KRT18
KRT19
KRT20
KRT23
KRT3
KRT4
KRT5
KRT6A
KRT6B
KRT6C
KRT7
KRT8

KRT81
KSR1
KSR2
KYNU
L1CAM
L2HGDH
L3MBTL1
L3MBTL2
L3MBTL3
LAG3
LAIR1
LAMA1
LAMA3
LAMA4
LAMB1
LAMB3
LAMC1
LAMC2
LAMP1
LAMP3
LAMTOR1
LANCL2
LAPTM4A
LAPTM4B
LAPTM5
LARP1
LARP7
LARS2
LAS1L
LASP1

LAT2
LATS1
LATS2
LBH
LCK
LCN2
LCOR
LCP1
LCP2
LDB1
LDHA
LDHB
LDHC
LDOC1
LECT1
LECT2
LEF1
LEMD3
LEO1
LEPREL1
LETMD1
LGALS1
LGALS3
LGALS3BP
LGALS4
LGALS7
LGALS8
LGALS9
LGI1
LGI3

LGMN
LGR4
LGR5
LGR6
LHCGR
LHFP
LIF
LIFR
LIG1
LIG3
LIG4
LILRA4
LILRB1
LILRB2
LILRB3
LILRB4
LIMA1
LIMD1
LIMK1
LIMK2
LIMS1
LIMS2
LIN28A
LIN28B
LIN9
LITAF
LLGL1
LLGL2
LMO1
LMO2

LMO3
LMO4
LMO7
LMTK2
LMTK3
LNX1
LONP1
LOX
LOXL2
LOXL3
LOXL4
LPA
LPAR1
LPAR2
LPAR3
LPAR4
LPAR5
LPCAT1
LPCAT2
LPHN2
LPXN
LRBA
LRG1
LRIG1
LRIG2
LRIG3
LRMP
LRP1
LRP12
LRP1B

LRP6
LRPPRC
LRRC15
LRRC26
LRRC32
LRRC3B
LRRC4
LRRC52
LRRC8A
LRRFIP1
LRRFIP2
LRRN1
LRRN3
LRSAM1
LRWD1
LSAMP
LSM7
LSP1
LST1
LTA
LTB
LTB4R
LTB4R2
LTBP2
LTBP4
LTBR
LTK
LUM
LXN
LY6D

LY6E
LY6K
LY75
LY9
LY96
LYAR
LYL1
LYN
LYNX1
LYPD3
LYPLA1
LYPLA2
LYVE1
LZTFL1
LZTR1
LZTS1
LZTS2
M6PR
MACC1
MACF1
MACROD2
MAD1L1
MAD2L1
MAD2L1BP
MAD2L2
MADCAM1
MAF
MAFB
MAFG
MAFK

MAGEA1
MAGEA10
MAGEA11
MAGEA12
MAGEA2
MAGEA3
MAGEA4
MAGEA6
MAGEA9
MAGEB1
MAGEB2
MAGEB3
MAGEC1
MAGEC2
MAGED1
MAGEL2
MAGI1
MAGI2
MAGI3
MAGOH
MAGT1
MAK
MAL2
MALT1
MAML1
MAML2
MAML3
MAN2A1
MAN2C1
MANBA

MAP1LC3A
MAP1LC3B
MAP1LC3C
MAP1S
MAP2K1
MAP2K2
MAP2K3
MAP2K4
MAP2K5
MAP2K6
MAP2K7
MAP3K1
MAP3K10
MAP3K11
MAP3K12
MAP3K14
MAP3K2
MAP3K3
MAP3K4
MAP3K5
MAP3K6
MAP3K7
MAP3K8
MAP3K9
MAP4K1
MAP4K3
MAP4K4
MAP4K5
MAP7
MAP9

MAPK1
MAPK10
MAPK11
MAPK12
MAPK13
MAPK14
MAPK3
MAPK4
MAPK6
MAPK7
MAPK8
MAPK8IP1
MAPK8IP2
MAPK8IP3
MAPK9
MAPKAP1
MAPKAPK2
MAPKAPK3
MAPKAPK5
MAPRE1
MAPRE2
MAPRE3
MARC1
MARCH5
MARCKS
MARCKSL1
MARK2
MARK3
MARK4
MARVELD1

MARVELD2
MARVELD3
MAST2
MASTL
MAT1A
MAT2A
MAT2B
MATR3
MAZ
MBD1
MBD2
MBD3
MBD4
MBIP
MC1R
MCAM
MCC
MCF2
MCF2L
MCL1
MCM2
MCM3
MCM3AP
MCM4
MCM5
MCM6
MCM7
MCM8
MCM9
MCPH1

MCRS1
MCTS1
MDC1
MDFI
MDK
MDM2
MDM4
ME1
MEA1
MECOM
MED1
MED12
MED13L
MED14
MED15
MED19
MED23
MED28
MED6
MEF2B
MEF2D
MEG3
MEIS1
MEIS2
MEIS3
MELK
MEMO1
MEN1
MEOX2
MERTK

MESDC2
METAP2
METTL14
METTL3
MFAP4
MFAP5
MFGE8
MFHAS1
MFI2
MFSD2A
MGAT3
MGAT5
MGAT5B
MGEA5
MGMT
MGST1
MIA
MIA2
MIA3
MIB1
MIB2
MICAL1
MICB
MIEN1
MIER1
MIF
MIIP
MINK1
MINPP1
MIP

MIPOL1
MIS12
MITF
MKI67
MKL1
MKL2
MKNK1
MKNK2
MKRN1
MLANA
MLC1
MLEC
MLF1
MLF2
MLH1
MLH3
MLKL
MLLT1
MLLT10
MLLT11
MLLT3
MLLT4
MLLT6
MLNR
MLST8
MLX
MLXIPL
MMAB
MMP1
MMP10

MMP11
MMP12
MMP13
MMP14
MMP15
MMP16
MMP17
MMP19
MMP2
MMP21
MMP23B
MMP24
MMP25
MMP26
MMP28
MMP3
MMP7
MMP9
MMS19
MN1
MNAT1
MNDA
MNX1
MOAP1
MOB1A
MOB1B
MOK
MORC2
MORC3
MORF4L1

MORF4L2
MOV10
MPC2
MPHOSPH9
MPL
MPO
MPP2
MPP3
MPPED2
MPZL1
MRC2
MRE11A
MRFAP1
MRGBP
MRGPRX2
MRPL19
MRPL23
MRPS30
MS4A1
MS4A3
MS4A7
MSH2
MSH3
MSH4
MSH5
MSH6
MSI1
MSI2
MSLN
MSMB

MSR1
MST1
MST1R
MST4
MT1A
MT1B
MT1E
MT1F
MT1G
MT1H
MT1M
MT1X
MT2A
MT3
MT4
MTA1
MTA2
MTA3
MTAP
MTBP
MTCP1
MTDH
MTHFD1
MTHFD2
MTHFR
MTHFS
MTMR3
MTOR
MTPN
MTRR

MTSS1
MTTP
MTUS1
MTX3
MUC1
MUC12
MUC13
MUC15
MUC16
MUC17
MUC2
MUC20
MUC3
MUC3A
MUC4
MUC5AC
MUC5B
MUC6
MUC7
MUCL1
MUM1
MUS81
MUT
MUTYH
MVD
MX2
MXD1
MXD3
MXD4
MXI1

MYADM
MYB
MYBBP1A
MYBL1
MYBL2
MYC
MYCBP
MYCBP2
MYCL
MYCN
MYCT1
MYD88
MYEOV
MYH11
MYL6
MYL9
MYO10
MYO18A
MYO18B
MYO1G
MYOD1
MYSM1
MYT1
MZF1
N4BP2
NAA10
NAA15
NAB1
NAB2
NACC1

NADSYN1
NAE1
NAF1
NAIF1
NAMPT
NANOG
NAP1L1
NAP1L4
NAPSA
NAT1
NAT10
NAT2
NAV3
NBL1
NBN
NBR1
NCAM1
NCEH1
NCF2
NCF4
NCK1
NCK2
NCKIPSD
NCOA1
NCOA2
NCOA3
NCOA4
NCOA5
NCOA6
NCOA7

NCOR1
NCOR2
NCR1
NCR2
NCR3
NCR3LG1
NDC1
NDC80
NDFIP1
NDN
NDOR1
NDRG1
NDRG2
NDRG3
NDRG4
NDUFA5
NDUFAF1
NDUFAF2
NDUFB7
NDUFB9
NDUFC2
NDUFS3
NEBL
NEDD1
NEDD4
NEDD4L
NEDD8
NEDD9
NEFH
NEFL

NEFM
NEIL1
NEIL2
NEIL3
NEK1
NEK11
NEK2
NEK3
NEK4
NEK6
NEK7
NEK9
NELL1
NELL2
NEMF
NENF
NEO1
NET1
NEU1
NEU2
NEU3
NEU4
NEUROD2
NEUROG1
NF1
NF2
NFAM1
NFATC1
NFATC2
NFATC2IP

NFE2
NFE2L1
NFE2L2
NFE2L3
NFIA
NFIB
NFIC
NFIL3
NFKB1
NFKB2
NFKBIA
NFKBIB
NFKBIE
NFKBIL1
NFKBIZ
NFRKB
NFX1
NFYA
NFYB
NFYC
NGFR
NGFRAP1
NHEJ1
NHLH1
NHLH2
NHP2
NHP2L1
NID1
NID2
NINJ1

NISCH
NKAIN2
NKD1
NKD2
NKG7
NKRF
NKTR
NLK
NLRC3
NLRC5
NLRP10
NLRP12
NLRP2
NLRP4
NLRP6
NLRP7
NMBR
NME1
NME5
NME6
NMI
NMRAL1
NMT2
NMU
NNAT
NNMT
NOB1
NOD1
NODAL
NOG

NOL3
NOM1
NONO
NOP10
NOP14
NOP2
NOS2
NOTCH1
NOTCH2
NOTCH3
NOTCH4
NOVA1
NOVA2
NOX1
NOX5
NOXA1
NOXO1
NPAT
NPM1
NPM3
NPRL2
NPRL3
NPTX1
NPTX2
NQO1
NQO2
NR0B1
NR0B2
NR1H4
NR1I2

NR1I3
NR2C1
NR2C2
NR2E1
NR2F1
NR2F2
NR2F6
NR4A1
NR4A2
NR4A3
NR5A1
NR5A2
NRARP
NRAS
NRBF2
NRBP1
NRCAM
NRF1
NRG2
NRG3
NRG4
NRIP1
NRK
NRM
NRP1
NRP2
NSD1
NSDHL
NSFL1C
NSL1

NSMAF
NSMCE2
NSUN2
NT5C2
NT5E
NTHL1
NTN1
NTN4
NTRK1
NTRK3
NTSR1
NUAK1
NUAK2
NUB1
NUBP1
NUCB1
NUCKS1
NUDC
NUDCD1
NUDT1
NUDT15
NUDT6
NUF2
NUFIP1
NUMA1
NUMB
NUMBL
NUP153
NUP205
NUP214

NUP50
NUP88
NUP98
NUPR1
NUSAP1
NUTF2
NXF1
NXF2
OAF
OAS1
OAS2
OASL
OAZ1
OBFC1
OCA2
OCIAD1
OCM
ODC1
ODF4
OGFR
OGG1
OIP5
OLA1
OLFM1
OLFM2
OLFM4
OLIG1
OLIG2
ONECUT1
ONECUT2

OPCML
OR51E1
OR51E2
ORAI1
ORAI2
ORAI3
ORAOV1
ORMDL3
OS9
OSBPL10
OSBPL2
OSBPL3
OSCAR
OSCP1
OSM
OSMR
OTOR
OTUB1
OTUD4
OTUD7B
OTULIN
OVCA2
OVOL1
OVOL2
OXCT1
OXER1
P2RY11
P2RY14
P2RY8
P4HA1

P4HA2
PA2G4
PABPC1
PACS1
PACS2
PAEP
PAF1
PAFAH1B2
PAFAH1B3
PAG1
PAGE1
PAGE4
PAICS
PAIP1
PAIP2
PAK1
PAK1IP1
PAK2
PAK4
PAK6
PAK7
PALB2
PALLD
PAPPA
PAPSS1
PAPSS2
PAQR3
PARD3
PARD6A
PARD6B

PARG
PARK7
PARL
PARP1
PARP14
PARP16
PARP2
PARP3
PARP4
PARP9
PARVB
PASD1
PATZ1
PAWR
PAX2
PAX3
PAX5
PAX7
PAX8
PAXIP1
PBK
PBLD
PBOV1
PBRM1
PBX1
PBX2
PBX3
PBXIP1
PCBP1
PCBP2

PCBP4
PCDH10
PCDH17
PCDH18
PCDH20
PCDH7
PCDH8
PCDH9
PCGF1
PCGF2
PCGF6
PCK2
PCM1
PCNA
PCNP
PCSK6
PCSK7
PCYT2
PDCD1
PDCD10
PDCD1LG2
PDCD2
PDCD4
PDCD5
PDCD6
PDCD6IP
PDCL3
PDE11A
PDE4B
PDE7A

PDE8B
PDGFA
PDGFB
PDGFC
PDGFD
PDGFRA
PDGFRB
PDGFRL
PDIA3
PDIA4
PDIA6
PDK1
PDLIM1
PDLIM2
PDLIM4
PDLIM5
PDLIM7
PDPK1
PDPN
PDRG1
PDS5A
PDS5B
PDXK
PDXP
PDZD2
PDZK1IP1
PEA15
PEAK1
PEBP1
PEBP4

PECAM1
PEG10
PEG3
PELI1
PELP1
PES1
PF4V1
PFDN5
PFKFB1
PFKFB2
PFKFB3
PFKFB4
PFKP
PFN1
PFN2
PGAM1
PGAM5
PGK1
PGLS
PGLYRP1
PGP
PGR
PGRMC1
PGRMC2
PHACTR2
PHACTR3
PHACTR4
PHB2
PHC1
PHC3

PHF1
PHF10
PHF12
PHF19
PHF2
PHF20
PHF3
PHF6
PHF8
PHGDH
PHIP
PHLDA1
PHLDA3
PHLDB1
PHLDB2
PHLPP1
PHLPP2
PHOX2B
PHTF1
PI15
PI16
PI3
PIAS1
PIAS2
PIAS3
PIAS4
PIBF1
PICALM
PIDD
PIFO

PIGF
PIH1D1
PIK3AP1
PIK3C2A
PIK3C2B
PIK3C2G
PIK3C3
PIK3CA
PIK3CB
PIK3CD
PIK3CG
PIK3IP1
PIK3R1
PIK3R2
PIK3R3
PIK3R4
PIK3R5
PIM1
PIM2
PIM3
PIN1
PINX1
PIP4K2A
PIP5K1A
PISD
PITPNB
PITPNM3
PIWIL1
PIWIL2
PIWIL3

PIWIL4
PJA1
PJA2
PKIB
PKIG
PKM
PKMYT1
PKN1
PKN3
PKNOX1
PKNOX2
PKP1
PKP3
PKP4
PLA2G16
PLA2G2A
PLA2G2D
PLA2G3
PLA2G4C
PLAC1
PLAG1
PLAGL1
PLAGL2
PLAU
PLAUR
PLCB1
PLCB3
PLCD4
PLCE1
PLCG1

PLCG2
PLD1
PLD2
PLEC
PLEK
PLEKHA2
PLEKHA3
PLEKHF1
PLEKHO1
PLK1
PLK2
PLK3
PLK4
PLOD2
PLOD3
PLP2
PLRG1
PLS3
PLSCR1
PLVAP
PLXDC1
PLXDC2
PLXNA1
PLXNB1
PLXNB3
PLXNC1
PLXND1
PMAIP1
PMEL
PMEPA1

PMF1
PML
PMS1
PMS2
PMS2CL
PNKP
PNPT1
PNRC1
PODXL
POGLUT1
POGZ
POLB
POLD1
POLD2
POLD3
POLDIP2
POLDIP3
POLE2
POLH
POLN
POLQ
POLR2A
POLR2C
POLR2E
POPDC3
PORCN
POSTN
POT1
POTEE
POU1F1

POU2AF1
POU2F1
POU2F2
POU2F3
POU3F2
POU3F3
POU4F1
POU4F2
POU5F1
POU6F1
POU6F2
PPAP2B
PPARGC1B
PPFIA1
PPFIBP1
PPHLN1
PPIA
PPIE
PPM1A
PPM1B
PPM1D
PPM1E
PPM1F
PPM1G
PPM1H
PPP1CA
PPP1CB
PPP1R10
PPP1R13B
PPP1R13L

PPP1R14C
PPP1R15A
PPP1R7
PPP1R8
PPP1R9A
PPP1R9B
PPP2CA
PPP2CB
PPP2R1A
PPP2R1B
PPP2R2A
PPP2R2B
PPP2R2C
PPP2R2D
PPP2R3A
PPP2R5A
PPP2R5B
PPP2R5C
PPP2R5E
PPP3CA
PPP4C
PPP5C
PPP6C
PRAF2
PRAM1
PRAME
PRB1
PRB2
PRB4
PRC1

PRCC
PRDM1
PRDM12
PRDM14
PRDM16
PRDM2
PRDM4
PRDM5
PRDM6
PRDX1
PRDX2
PRDX3
PRDX4
PRDX6
PREB
PREX1
PREX2
PRF1
PRG2
PRG3
PRICKLE1
PRICKLE2
PRIM1
PRIMA1
PRKAA1
PRKAA2
PRKACA
PRKACB
PRKAR1A
PRKAR2A

PRKAR2B
PRKCA
PRKCB
PRKCD
PRKCDBP
PRKCE
PRKCI
PRKCQ
PRKCSH
PRKCZ
PRKD1
PRKD2
PRKDC
PRKRA
PRL
PRLR
PRM3
PRMT1
PRMT2
PRMT3
PRMT5
PRMT6
PRMT7
PRMT8
PROCR
PROK1
PROKR1
PROM1
PROM2
PROX1

PRPF19
PRPF4
PRPF4B
PRPF6
PRPS2
PRR13
PRRC2A
PRRX1
PRRX2
PRSS1
PRSS2
PRSS21
PRSS3
PRSS8
PRTN3
PRUNE2
PSAT1
PSCA
PSG5
PSIP1
PSMA1
PSMA2
PSMA3
PSMA4
PSMA5
PSMA7
PSMB1
PSMB10
PSMB11
PSMB2

PSMB3
PSMB4
PSMB5
PSMB6
PSMB7
PSMC1
PSMC2
PSMC3IP
PSMC5
PSMD1
PSMD10
PSMD14
PSMD2
PSMD3
PSMD4
PSMD7
PSMD9
PSME1
PSME2
PSME3
PSMF1
PSMG1
PSPH
PSTPIP1
PTAFR
PTBP1
PTBP2
PTBP3
PTCH1
PTCH2

PTCRA
PTEN
PTENP1
PTER
PTGER2
PTGER4
PTGES
PTGES2
PTGFRN
PTGIS
PTGR1
PTGS1
PTGS2
PTH2
PTHLH
PTK2
PTK6
PTK7
PTN
PTOV1
PTP4A1
PTP4A2
PTP4A3
PTPMT1
PTPN1
PTPN11
PTPN12
PTPN13
PTPN14
PTPN18

PTPN2
PTPN21
PTPN23
PTPN3
PTPN4
PTPN6
PTPN7
PTPN9
PTPRA
PTPRB
PTPRC
PTPRCAP
PTPRD
PTPRE
PTPRF
PTPRG
PTPRH
PTPRJ
PTPRK
PTPRM
PTPRN2
PTPRO
PTPRR
PTPRS
PTPRT
PTPRU
PTPRZ1
PTRF
PTTG1
PTTG1IP

PUF60
PUM1
PVRL1
PVRL2
PVRL3
PVRL4
PXDN
PXN
PXT1
PYCARD
PYGB
PYGM
PYGO1
PYGO2
PYHIN1
QKI
QPCT
QSOX1
RAB11A
RAB11FIP1
RAB1A
RAB20
RAB21
RAB22A
RAB23
RAB25
RAB26
RAB27A
RAB27B
RAB2A

RAB30
RAB31
RAB33A
RAB34
RAB36
RAB37
RAB39A
RAB4B
RAB5C
RAB6B
RAB6C
RABEP1
RABGAP1
RABGAP1L
RAC1
RAC2
RAC3
RACGAP1
RAD1
RAD17
RAD18
RAD21
RAD23A
RAD23B
RAD50
RAD51
RAD51AP1
RAD51B
RAD51C
RAD51D

RAD52
RAD54B
RAD54L
RAD9A
RAE1
RAET1E
RAET1G
RAET1L
RAF1
RAG2
RALA
RALB
RALBP1
RALGDS
RALY
RANBP17
RANBP2
RAP1A
RAP1B
RAP1GAP
RAP1GDS1
RAP2A
RAP2B
RAPGEF1
RAPGEF2
RAPGEF3
RAPGEF6
RAPH1
RARA
RARB

RARG
RARRES1
RARRES2
RARRES3
RARS
RASA1
RASA2
RASA3
RASA4
RASAL1
RASAL2
RASD1
RASGEF1A
RASGRF1
RASGRF2
RASGRP1
RASGRP2
RASGRP3
RASGRP4
RASIP1
RASSF1
RASSF10
RASSF2
RASSF3
RASSF4
RASSF5
RASSF6
RASSF7
RASSF8
RB1

RB1CC1
RBAK
RBBP4
RBBP5
RBBP6
RBBP7
RBBP8
RBBP9
RBCK1
RBFOX2
RBFOX3
RBL1
RBL2
RBM10
RBM14
RBM15
RBM17
RBM25
RBM3
RBM38
RBM4
RBM5
RBM6
RBM8A
RBMS3
RBMX
RBP1
RBP2
RBP3
RBPJ

RBX1
RC3H1
RCC2
RCE1
RCHY1
RCN2
RCOR1
RCSD1
RCVRN
RDH11
RDM1
RECK
RECQL
RECQL4
RECQL5
REG1A
REG1B
REG3A
REG3G
REG4
REL
RELA
RELB
RELT
REPIN1
REPS2
RERE
RERG
RET
RETN

REV1
REV3L
RFC1
RFC2
RFC3
RFC4
RFC5
RFWD2
RFX1
RFX5
RGMB
RGS1
RGS12
RGS13
RGS17
RGS19
RGS5
RHBDD1
RHBDF2
RHBDL2
RHEB
RHEBL1
RHOA
RHOB
RHOBTB1
RHOBTB2
RHOBTB3
RHOC
RHOH
RHOJ

RHOU
RHOV
RHOXF2
RHPN2
RICTOR
RIF1
RIN1
RING1
RINT1
RIOK3
RIPK1
RIPK2
RIPK3
RIPK4
RIT1
RLN2
RMI1
RMI2
RNASE1
RNASE2
RNASEH1
RNASEH2A
RNASEL
RNASET2
RND1
RND2
RND3
RNF10
RNF11
RNF111

RNF115
RNF13
RNF138
RNF139
RNF14
RNF146
RNF168
RNF17
RNF19A
RNF2
RNF20
RNF216
RNF31
RNF34
RNF4
RNF40
RNF41
RNF43
RNF5
RNF6
RNF7
RNF8
RNGTT
RNH1
RNMT
ROBO1
ROBO4
ROCK1
ROCK2
ROMO1

ROPN1
ROR1
ROR2
RORA
ROS1
RP9
RPA1
RPA2
RPAP2
RPAP3
RPL10A
RPL11
RPL12
RPL13
RPL15
RPL19
RPL22
RPL23A
RPL24
RPL26
RPL27
RPL27A
RPL29
RPL30
RPL35A
RPL36A
RPL37
RPL37A
RPL38
RPL39

RPL41
RPL5
RPL6
RPL7A
RPLP0
RPLP1
RPLP2
RPN1
RPN2
RPRM
RPS14
RPS15A
RPS20
RPS23
RPS24
RPS25
RPS27
RPS27A
RPS29
RPS3A
RPS4Y1
RPS6
RPS6KA1
RPS6KA2
RPS6KA4
RPS6KA5
RPS6KA6
RPS6KB1
RPS6KB2
RPS9

RPSA
RPTOR
RQCD1
RRAD
RRAGA
RRAGC
RRAGD
RRAS
RRAS2
RREB1
RRH
RRM1
RRM2
RRM2B
RRN3
RRP12
RRP1B
RSF1
RSL1D1
RSPO1
RSPO2
RSPO3
RSU1
RTCA
RTEL1
RTKN
RTN1
RTN4
RUNX1
RUNX1T1

RUNX2
RUNX3
RUVBL1
RUVBL2
RXRA
RYBP
RYK
S100A1
S100A10
S100A11
S100A13
S100A14
S100A16
S100A2
S100A3
S100A4
S100A5
S100A6
S100A7
S100A7A
S100A8
S100A9
S100P
S1PR1
S1PR2
S1PR3
SAA1
SAA4
SAE1
SAFB

SAFB2
SALL2
SALL3
SALL4
SAMD4A
SAMD9
SAMD9L
SAMSN1
SAP130
SAP30
SART1
SART3
SASH1
SASS6
SAT2
SATB1
SATB2
SAV1
SBDS
SBF1
SBNO2
SCAND1
SCARA3
SCARA5
SCEL
SCG2
SCG3
SCG5
SCGB1D2
SCGB2A1

SCGB2A2
SCGB3A1
SCGB3A2
SCGN
SCHIP1
SCML2
SCRIB
SCRN1
SCT
SCTR
SCUBE2
SCUBE3
SCYL1
SDC1
SDC2
SDC4
SDCBP
SDCCAG8
SDF2
SDF4
SDHA
SDHAF1
SDHAF2
SDHB
SDHC
SDHD
SDK1
SEC14L1
SEC14L2
SEC23IP

SEC61A1
SEC61B
SEC61G
SECTM1
SEL1L
SELENBP1
SEMA3B
SEMA3C
SEMA3E
SEMA3F
SEMA3G
SEMA4B
SEMA4C
SEMA4D
SEMA4F
SEMA5A
SEMA6B
SEMA6D
SENP1
SENP2
SENP3
SENP5
SENP6
SENP7
SENP8
SEPP1
SEPT1
SEPT10
SEPT11
SEPT2

SEPT4
SEPT6
SEPT7
SEPT8
SEPT9
SEPW1
SERBP1
SERINC3
SERP2
SERPINA3
SERPINA9
SERPINB1
SERPINB10
SERPINB11
SERPINB13
SERPINB2
SERPINB3
SERPINB4
SERPINB5
SERPINB8
SERPINB9
SERPINE1
SERPINE2
SERPINF1
SERPINH1
SERPINI1
SERPINI2
SERTAD1
SESN1
SESN2

SESN3
SETBP1
SETD1A
SETD1B
SETD2
SETD7
SETD8
SETDB1
SETMAR
SEZ6L
SF3A1
SF3B1
SF3B2
SF3B3
SFMBT1
SFMBT2
SFN
SFPQ
SFRP1
SFRP2
SFRP4
SFRP5
SFTPA2
SGCA
SGK3
SGMS1
SGOL1
SGOL2
SGPL1
SGPP1

SGPP2
SGTA
SH2D1A
SH2D1B
SH2D3C
SH2D4A
SH3BGRL3
SH3BP2
SH3BP5
SH3GL1
SH3GL2
SH3GL3
SH3GLB1
SH3KBP1
SH3PXD2A
SH3RF1
SHARPIN
SHBG
SHC1
SHC3
SHC4
SHE
SHFM1
SHH
SHMT1
SHMT2
SHOC2
SHPRH
SHQ1
SHROOM2

SIAE
SIAH1
SIAH2
SIGIRR
SIGLEC10
SIGLEC14
SIGLEC5
SIGLEC7
SIGLEC9
SIK1
SIK2
SIK3
SIM2
SIN3A
SIN3B
SIPA1
SIRPA
SIRPB1
SIRT1
SIRT2
SIRT3
SIRT4
SIRT5
SIRT6
SIRT7
SIVA1
SIX1
SKA1
SKA2
SKA3

SKAP1
SKAP2
SKI
SKIL
SKOR1
SKP1
SKP2
SLAMF1
SLAMF6
SLAMF7
SLC12A5
SLC15A3
SLC16A12
SLC16A3
SLC16A4
SLC17A3
SLC18A1
SLC19A1
SLC19A2
SLC1A5
SLC20A1
SLC22A1
SLC22A11
SLC22A16
SLC22A17
SLC22A18
SLC22A3
SLC24A4
SLC24A5
SLC25A10

SLC25A23
SLC25A37
SLC27A2
SLC28A1
SLC28A3
SLC29A1
SLC29A2
SLC2A1
SLC2A14
SLC2A4RG
SLC30A9
SLC31A1
SLC31A2
SLC34A2
SLC35B2
SLC38A2
SLC38A3
SLC39A1
SLC39A10
SLC39A2
SLC39A6
SLC39A7
SLC39A9
SLC3A2
SLC43A1
SLC44A1
SLC45A2
SLC45A3
SLC46A1
SLC4A3

SLC4A7
SLC52A3
SLC5A5
SLC5A8
SLC6A14
SLC7A11
SLC7A4
SLC7A5
SLC7A6
SLC7A8
SLC9A1
SLC9A3R1
SLCO1A2
SLCO1B1
SLCO1B3
SLCO2B1
SLCO3A1
SLCO4A1
SLCO4C1
SLFN11
SLIRP
SLIT1
SLIT2
SLIT3
SLK
SLN
SLPI
SLURP1
SLX4
SMAD1

SMAD2
SMAD3
SMAD4
SMAD5
SMAD6
SMAD7
SMAP1
SMAP2
SMARCA1
SMARCA2
SMARCA4
SMARCA5
SMARCAD1
SMARCAL1
SMARCB1
SMARCC1
SMARCC2
SMARCD1
SMARCD3
SMARCE1
SMC1A
SMC1B
SMC3
SMC4
SMC5
SMC6
SMCHD1
SMG1
SMO
SMOC2

SMOX
SMPD1
SMPD3
SMR3B
SMUG1
SMURF1
SMURF2
SMYD2
SMYD3
SNAI1
SNAI2
SNAI3
SNCG
SND1
SNF8
SNIP1
SNRPD3
SNRPE
SNTN
SNW1
SNX10
SNX16
SNX19
SOBP
SOCS1
SOCS2
SOCS3
SOCS4
SOCS5
SOCS6

SOCS7
SOD2
SORBS1
SORBS2
SOS1
SOSTDC1
SOX1
SOX10
SOX11
SOX12
SOX17
SOX18
SOX2
SOX21
SOX30
SOX4
SOX5
SOX7
SP1
SP100
SP110
SP140
SP2
SP3
SPA17
SPACA3
SPAG5
SPAG6
SPAG8
SPAG9

SPANXA1
SPANXC
SPARC
SPARCL1
SPATA13
SPATA18
SPDEF
SPDYA
SPEN
SPG20
SPHK1
SPHK2
SPIB
SPINK1
SPINK7
SPINT1
SPINT2
SPN
SPNS2
SPOCK1
SPOCK2
SPOCK3
SPON1
SPON2
SPOP
SPP1
SPPL2A
SPRED1
SPRED2
SPRR1A

SPRR1B
SPRR2A
SPRR2D
SPRR3
SPRY1
SPRY2
SPRY3
SPRY4
SPTAN1
SPTBN1
SPZ1
SQLE
SQSTM1
SRA1
SRC
SRCAP
SRD5A1
SRD5A2
SRGAP1
SRGAP2
SRGN
SRMS
SRPK1
SRPK2
SRPX
SRPX2
SRRM1
SRRM2
SRRT
SRSF1

SRSF10
SRSF2
SRSF3
SRSF4
SRSF5
SRSF6
SRXN1
SS18
SS18L1
SSBP1
SSBP2
SSH2
SSPN
SSR1
SSR2
SSRP1
SSTR1
SSTR2
SSTR3
SSTR4
SSTR5
SSX1
SSX2
SSX2IP
SSX4
ST13
ST14
ST3GAL1
ST3GAL2
ST3GAL3

ST3GAL4
ST3GAL5
ST3GAL6
ST6GAL1
ST6GALNAC1
ST6GALNAC2
ST6GALNAC5
ST7L
ST8SIA1
ST8SIA2
ST8SIA4
STAB1
STAG1
STAG2
STAG3
STAP2
STARD10
STARD13
STARD3
STARD5
STARD7
STAT1
STAT2
STAT3
STAT5A
STAT5B
STAT6
STC1
STC2
STEAP1

STEAP2
STEAP3
STEAP4
STIL
STIM1
STIM2
STIP1
STK10
STK11
STK11IP
STK16
STK17A
STK17B
STK24
STK25
STK3
STK31
STK33
STK36
STK38
STK38L
STK4
STMN1
STMN3
STOML1
STOML2
STOX1
STRA13
STRN
STRN3

STT3A
STT3B
STUB1
STX11
STX17
STX6
STX8
STXBP2
STXBP4
STXBP6
STYK1
SUFU
SULF1
SULF2
SULT1A1
SULT1A2
SULT1A3
SULT1C2
SULT1E1
SULT2B1
SUMO1
SUMO2
SUMO3
SUPT16H
SUPT3H
SUPT5H
SUPT6H
SURF4
SURF6
SUSD2

SUV39H1
SUV39H2
SUV420H1
SUV420H2
SUZ12
SVEP1
SVIL
SWAP70
SYF2
SYK
SYMPK
SYNCRIP
SYNE1
SYNJ2
SYNJ2BP
SYNPO2
SYT9
SYVN1
T
TAB1
TAB2
TAB3
TAC1
TAC4
TACC1
TACC2
TACC3
TACSTD2
TADA2A
TADA3

TAF12
TAF15
TAF1A
TAF1B
TAF1C
TAF3
TAF4
TAF4B
TAF7
TAF9
TAGLN
TAGLN2
TAL1
TAL2
TALDO1
TAP1
TAP2
TARBP1
TARBP2
TAX1BP1
TAX1BP3
TAZ
TBC1D3
TBC1D7
TBCB
TBK1
TBL1X
TBL1XR1
TBPL2
TBX19

TBX2
TBX3
TBXAS1
TCEA1
TCEAL1
TCEAL7
TCEB1
TCEB2
TCF12
TCF20
TCF21
TCF3
TCF4
TCF7
TCF7L1
TCF7L2
TCFL5
TCHP
TCL1A
TCL1B
TCP1
TCRA
TCTN1
TDG
TDGF1
TDP1
TDP2
TDRD1
TEAD1
TEAD2

TEAD3
TEAD4
TENC1
TEP1
TERF1
TERF2
TERF2IP
TET1
TET2
TET3
TEX14
TFAP2A
TFAP2B
TFAP2C
TFAP2E
TFAP4
TFCP2
TFCP2L1
TFDP1
TFDP2
TFE3
TFEB
TFEC
TFF1
TFF2
TFF3
TFG
TFPI2
TFPT
TFRC

TGDS
TGFA
TGFB1
TGFB1I1
TGFB2
TGFBI
TGFBR1
TGFBR2
TGFBR3
TGFBRAP1
TGIF1
TGIF2
TGM2
TGM3
TGM4
THADA
THAP11
THBD
THBS1
THBS2
THBS3
THEM4
THOC1
THOC5
THRA1
THRAP3
THRB
THSD4
THY1
TIA1

TIAF1
TIAL1
TIAM1
TIAM2
TICAM1
TICAM2
TICRR
TIE1
TIFA
TIGIT
TIMD4
TIMP1
TIMP2
TIMP3
TIMP4
TINAGL1
TINF2
TIPARP
TIPIN
TJP1
TK1
TK2
TKT
TKTL1
TLE1
TLE2
TLE3
TLE4
TLK1
TLK2

TLN1
TLN2
TLR1
TLR10
TLR2
TLR3
TLR4
TLR5
TLR6
TLR7
TLR8
TLR9
TLX1
TLX3
TM4SF1
TM4SF4
TM4SF5
TM7SF2
TM9SF4
TMBIM4
TMBIM6
TMC6
TMC8
TMEFF1
TMEFF2
TMEM100
TMEM127
TMEM158
TMEM176B
TMEM30A

TMEM30B
TMEM45A
TMEM66
TMEM8B
TMEM97
TMF1
TMPRSS11A
TMPRSS13
TMPRSS2
TMPRSS3
TMPRSS4
TMSB10
TMSB4X
TMX2
TNF
TNFAIP1
TNFAIP2
TNFAIP3
TNFAIP6
TNFAIP8
TNFAIP8L2
TNFRSF10A
TNFRSF10B
TNFRSF10C
TNFRSF10D
TNFRSF11A
TNFRSF11B
TNFRSF12A
TNFRSF13B
TNFRSF13C

TNFRSF14
TNFRSF17
TNFRSF18
TNFRSF19
TNFRSF1A
TNFRSF1B
TNFRSF21
TNFRSF25
TNFRSF4
TNFRSF6B
TNFRSF8
TNFRSF9
TNFSF10
TNFSF11
TNFSF12
TNFSF13
TNFSF13B
TNFSF14
TNFSF15
TNFSF18
TNFSF4
TNFSF8
TNFSF9
TNIK
TNIP1
TNIP2
TNIP3
TNK1
TNK2
TNKS

TNKS2
TNN
TNPO2
TNPO3
TNR
TNRC6A
TNRC6B
TNS1
TNS3
TNS4
TOB1
TOB2
TOM1L1
TOM1L2
TOMM34
TONSL
TOP1
TOP1MT
TOP2A
TOP2B
TOP3A
TOP3B
TOPBP1
TOPORS
TOX3
TOX4
TP53
TP53BP1
TP53BP2
TP53I11

TP53I3
TP53INP1
TP53INP2
TP53RK
TP63
TP73
TPBG
TPD52
TPD52L1
TPD52L2
TPM1
TPM4
TPMT
TPO
TPP1
TPP2
TPT1
TPTE
TPX2
TRA2B
TRAC
TRADD
TRAF1
TRAF2
TRAF3
TRAF3IP1
TRAF3IP2
TRAF4
TRAF5
TRAF6

TRAF7
TRAIP
TRAM1
TRAM2
TRAP1
TRAPPC9
TRDMT1
TREM1
TREML2
TRERF1
TREX2
TRIB1
TRIB2
TRIB3
TRIM13
TRIM16
TRIM17
TRIM2
TRIM22
TRIM24
TRIM25
TRIM27
TRIM28
TRIM29
TRIM3
TRIM32
TRIM33
TRIM35
TRIM36
TRIM37

TRIM39
TRIM44
TRIM5
TRIM59
TRIM6
TRIM62
TRIM8
TRIM9
TRIP10
TRIP11
TRIP12
TRIP13
TRIP6
TRIT1
TROAP
TRPC4AP
TRPM1
TRPM2
TRPM7
TRPS1
TRRAP
TSC1
TSC2
TSC22D1
TSC22D3
TSG101
TSGA10
TSPAN1
TSPAN13
TSPAN2

TSPAN3
TSPAN31
TSPAN32
TSPAN33
TSPAN4
TSPAN5
TSPAN7
TSPAN8
TSPY1
TSPYL2
TSPYL5
TSR2
TSSC4
TSTA3
TTC4
TTF1
TTF2
TTI1
TTK
TTYH1
TUBA1B
TUBB
TUBB1
TUBB2A
TUBB3
TUBG1
TUBGCP2
TUFM
TUSC2
TUSC3

TUSC5
TWF1
TWF2
TWIST1
TWIST2
TWSG1
TXLNA
TXN
TXN2
TXNDC17
TXNDC5
TXNIP
TXNL1
TXNRD1
TXNRD2
TYK2
TYMP
TYMS
TYRO3
TYROBP
TYRP1
U2AF1
U2AF2
UACA
UAP1
UBA1
UBA3
UBA6
UBAP1
UBD

UBE2A
UBE2B
UBE2C
UBE2D1
UBE2D2
UBE2D3
UBE2E1
UBE2I
UBE2L3
UBE2L6
UBE2M
UBE2N
UBE2Q1
UBE2Q2
UBE2S
UBE2T
UBE2V1
UBE2V2
UBE3A
UBE3C
UBE4A
UBE4B
UBIAD1
UBN1
UBQLN1
UBQLN4
UBR2
UBR3
UBR4
UBR5

UCHL1
UCHL5
UCK1
UCK2
UCN
UFC1
UFL1
UGCG
UGDH
UGT1A1
UGT1A10
UGT1A5
UGT1A6
UGT1A7
UGT1A8
UGT1A9
UGT2B10
UGT2B11
UGT2B15
UGT2B17
UGT2B28
UGT2B4
UHMK1
UHRF1
UHRF2
ULBP1
ULBP2
ULBP3
ULK1
ULK2

ULK3
ULK4
UNC13D
UNC45A
UNC5A
UNC5B
UNC5C
UNC5D
UNG
UPK1A
UPK1B
UPK2
UPK3A
UPP1
UPRT
UQCRB
UQCRFS1
UQCRH
URAHP
URGCP
URI1
USF1
USF2
USMG5
USP1
USP10
USP11
USP12
USP13
USP14

USP15
USP16
USP17L2
USP18
USP19
USP2
USP20
USP21
USP22
USP24
USP28
USP29
USP3
USP33
USP34
USP36
USP37
USP39
USP4
USP42
USP44
USP46
USP47
USP5
USP6
USP7
USP8
USP9X
UTF1
UTP20

UTP6
UTY
UVRAG
UVSSA
UXT
VAC14
VANGL1
VARS2
VASH1
VASH2
VASN
VAV1
VAV2
VAV3
VBP1
VCAM1
VCAN
VDAC1
VDR
VEGFA
VEGFB
VEGFC
VENTX
VEZF1
VEZT
VGLL3
VGLL4
VHL
VIL1
VIMP

VIPR1
VMP1
VOPP1
VPRBP
VPREB3
VPS25
VPS33A
VPS37A
VPS39
VPS4B
VPS53
VRK1
VRK2
VSIG4
VTCN1
VWA2
VWA5A
WAPAL
WASF1
WASF2
WASF3
WASL
WBP2
WBSCR22
WDHD1
WDR1
WDR12
WDR48
WDR5
WDR77

WDR82
WEE1
WFDC1
WFDC2
WHSC1
WHSC1L1
WIF1
WIPF1
WIPI1
WIPI2
WISP1
WISP2
WISP3
WNK2
WNT1
WNT10A
WNT10B
WNT11
WNT16
WNT2
WNT2B
WNT3
WNT3A
WNT4
WNT5A
WNT5B
WNT6
WNT7A
WNT7B
WNT9A

WRAP53
WRN
WSB1
WT1
WTAP
WTIP
WWC1
WWOX
WWP1
WWP2
WWTR1
XAB2
XAF1
XBP1
XCL1
XCL2
XCR1
XIAP
XPA
XPC
XPO1
XPO4
XPO5
XPR1
XRCC1
XRCC2
XRCC3
XRCC4
XRCC5
XRCC6

YAF2
YAP1
YARS
YBX1
YEATS4
YES1
YOD1
YTHDF2
YWHAB
YWHAE
YWHAG
YWHAH
YWHAQ
YWHAZ
YY1
YY1AP1
ZAP70
ZBP1
ZBTB10
ZBTB16
ZBTB17
ZBTB20
ZBTB33
ZBTB38
ZBTB4
ZBTB46
ZBTB7A
ZBTB7B
ZBTB7C
ZC3H12A

ZC3H12D
ZC3HAV1
ZCCHC11
ZCCHC6
ZDHHC2
ZDHHC9
ZEB1
ZEB2
ZFAND2A
ZFHX3
ZFP36
ZFP36L1
ZFP36L2
ZFP42
ZFP64
ZFPM1
ZFPM2
ZFYVE1
ZFYVE16
ZFYVE20
ZG16
ZHX1
ZHX2
ZIC1
ZIC4
ZMIZ1
ZMYM2
ZMYM3
ZMYND10
ZMYND11

ZMYND8
ZNF10
ZNF131
ZNF133
ZNF143
ZNF146
ZNF148
ZNF165
ZNF185
ZNF217
ZNF224
ZNF24
ZNF268
ZNF281
ZNF300
ZNF331
ZNF335
ZNF350
ZNF354A
ZNF354C
ZNF365
ZNF366
ZNF382
ZNF384
ZNF395
ZNF423
ZNF521
ZNF652
ZNF703
ZNF750

ZNF76
ZNF91
ZNRD1
ZNRF3
ZRANB3
ZRSR2
ZWILCH
ZWINT
ZYX

Suppl. Tab. 4. Somatic mutations identified in 23 CP and 10 BC CML patients by whole exome sequencing. * indicates a stop codon. The "Mutation Ratio" label indicates the proportion of total reads that were mutated. Reference human genome is GRCh37 (hg19). Variant positions are reported using a +1 notation.

Disease Phase	Sample ID	Mutation	Amino acid	Coordinates	Base change	Gene Ranker	OncoScore	Mutation Ratio
CP	CMLPh+001	PATZ1	F539L	chr22:31724801	G/T	2.00	66.94	47.83
CP	CMLPh+002	KBTBD7	I196V	chr13:41767808	T/C	0.00	0.00	52.46
CP		ASXL1	Q829*	chr20:31023000	C/T	0.00	77.41	36.00
CP		MAP3K4	R433*	chr6:161470601	C/T	2.00	39.91	54.17
CP	CMLPh+003	HMCN1	C1241Y	chr1:185964163	G/A	0.00	16.54	38.81
CP		SPHKAP	N1027K	chr2:228882489	G/T	3.00	0.00	38.24
CP		TMED4	G76D	chr7:44621356	C/T	0.00	0.00	45.28
CP		PTPRD	L760F	chr9:8460424	G/A	4.00	49.33	39.29
CP	CMLPh+004	DLG5	V924I	chr10:79572041	C/T	0.00	63.85	55.56
CP		TEP1	K1981R	chr14:20845585	T/C	1.00	58.88	53.06
CP		C17orf53	A247V	chr17:42225911	C/T	0.00	0.00	37.66
CP		C1orf62	A281V	chr1:109394445	G/A	0.00	0.00	48.57

CP		SCN10A	I1482T	chr3:38743542	A/G	0.00	7.24	50.00
CP		POLN	F475L	chr4:2082722	A/G	2.00	37.50	54.65
CP		AKAP4	A579V	chrX:49957628	G/A	2.00	28.60	47.83
CP	CMLPh+005	AC013553.1 (SLC51B)	I50R	chr15:65343904	T/G	0.00	0.00	54.17
CP		HSF2	Q375R	chr6:122744779	A/G	0.00	24.95	47.62
CP		CBWD6	C239Y	chr9:69238176	C/T	0.00	0.00	39.18
CP		MYOF	Q177P	chr10:95169400	T/G	0.00	20.87	47.56
CP		PLXNB1	R825H	chr3:48459884	C/T	0.00	39.08	53.57
CP	CMLPh+006	POU6F1	D140G	chr12:51585526	T/C	0.00	23.71	56.41
CP	CMLPh+007	TRAFD1	V526M	chr12:112589901	G/A	0.00	0.00	45.16
CP		TCEB3B	A576T	chr18:44559910	C/T	0.00	0.00	45.83
CP		IGSF21	H279Q	chr1:18692013	C/G	0.00	0.00	47.71
CP		LIMS2	F138C	chr2:128412016	A/C	0.00	24.14	52.17
CP	CMLPh+008	TUBA1B	I115V	chr12:49523057	T/C	0.00	30.04	52.17
CP		RBM19	H512R	chr12:114383724	T/C	0.00	0.00	48.08
CP		ASB2	V173M	chr14:94417564	C/T	0.00	25.84	45.83

CP		OR10H2	V65I	chr19:15839046	G/A	0.00	0.00	51.27
CP		ROR2	A643T	chr9:94486849	C/T	3.00	29.51	51.83
CP	CMLPh+010	-	-	-	-	-	-	-
CP	CMLPh+011	ARNTL	K259N	chr12:13391271	G/C	0.00	12.94	44.76
CP		MYO16	R747H	chr13:109617187	G/A	1.00	0.00	58.82
CP		KARS	A54P	chr16:75675524	C/G	1.00	17.39	43.04
CP		PCDHA5	R12W	chr5:140201394	C/T	0.00	0.00	45.45
CP		SH3PXD2B	A566T	chr5:171766413	C/T	0.00	11.23	43.59
CP		STC2	R225C	chr5:172745086	G/A	0.00	50.49	45.00
CP		DUSP22	A56V	chr6:335142	C/T	1.00	55.35	32.65
CP		GPER1	D202N	chr7:1131968	G/A	0.00	36.20	31.82
CP		BX255925.17-2	W365*	chr9:140147839	G/A	0.00	0.00	71.88
CP	CMLPh+012	KAT7	R311G	chr17:47893243	C/G	2.00	31.60	46.55
CP	CMLPh+013	UNC13C	R626Q	chr15:54786821	G/A	0.00	9.92	50.55
CP		BRSK1	R701Q	chr19:55820019	G/A	1.25	39.31	39.13
CP		CACNA1I	I226V	chr22:40030665	A/G	1.50	9.39	46.40

CP		AR	K659N	chrX:66931335	G/T	6.25	15.88	46.02
CP	CMLPh+014	NBAS	I1685V	chr2:15427282	T/C	0.00	14.52	42.55
CP	CMLPh+015	HNRNPH3	Y156C	chr10:70098927	A/G	0.00	0.00	51.38
CP		CADM1	A262V	chr11:115088648	G/A	1.25	66.31	51.22
CP		OR11G2	R160P	chr14:20665973	G/C	0.00	0.00	51.11
CP		TINAGL1	V443I	chr1:32052503	G/A	0.00	42.13	50.00
CP		LEPR	S882F	chr1:66088636	C/T	1.00	2.85	45.45
CP		TNNI3K	M717L	chr1:74954900	A/C	2.00	1.90	53.42
CP		FCAMR	G374*	chr1:207133966	C/A	0.00	0.00	39.73
CP		CCDC80	S648I	chr3:112349052	C/A	1.00	50.22	52.50
CP		GPR156	A705T	chr3:119886211	C/T	0.00	0.00	48.48
CP		CTNNA1	N257S	chr5:138160400	A/G	4.00	64.38	54.35
CP		JAKMIP2	D409H	chr5:147021327	C/G	1.00	0.00	43.42
CP	CMLPh+016	SPRED3	P159L	chr19:38885335	C/T	0.00	0.00	57.45
CP		VN1R1	S199N	chr19:57967259	C/T	1.00	8.89	47.62
CP		DSCAM	V1954M	chr21:41385098	C/T	0.00	8.95	55.88

CP		AMACR	E173K	chr5:34024078	C/T	1.00	82.50	48.17
CP		CYLN2	K39E	chr7:73731991	A/G	0.00	19.84	40.38
CP	CMLPh+017	MYH7	A161T	chr14:23901869	C/T	0.00	4.05	50.00
CP		BBS2	G224D	chr16:56540078	C/T	0.00	4.17	51.00
CP		WDR63	V807I	chr1:85595682	G/A	0.00	0.00	40.96
CP		SHPRH	R936C	chr6:146256227	G/A	0.00	31.10	49.02
CP		QKI	D74V	chr6:163876389	A/T	2.00	35.13	46.32
CP		IFNA8	A2T	chr9:21409179	G/A	0.00	32.19	32.50
CP	CMLPh+018	MARK3	R573*	chr14:103958244	C/T	1.00	23.15	56.16
CP		CMIP	A730V	chr16:81739201	C/T	0.00	0.00	43.75
CP		DDX4	R596H	chr5:55110902	G/A	2.00	17.39	55.56
CP		TINAG	G54E	chr6:54173509	G/A	0.00	16.97	40.38
CP	CMLPh+019	RMDN2	R428I	chr2:38208444	G/T	0.00	0.00	53.09
CP		SORCS2	E671Q	chr4:7725526	G/C	0.00	14.14	51.47
CP		SOX8	A306V	chr16:1034962	C/T	0.00	12.85	52.17
CP	CMLPh+020	TEX37	P7S	chr2:88825181	C/T	0.00	0.00	60.71

CP		TRA2B	R65Q	chr3:185643391	C/T	0.00	26.72	64.52
CP		MSMO1	P111L	chr4:166259017	C/T	1.00	21.23	52.98
CP		GFRAL	C210W	chr6:55216310	T/G	0.00	0.00	48.51
CP		SVEP1	T523M	chr9:113261434	G/A	0.00	37.50	47.87
CP		ARHGAP23	I1002M	chr17:36646362	C/G	0.00	0.00	44.95
CP		C19orf54	R110*	chr19:41251099	G/A	0.00	0.00	44.87
CP	CMLPh+021	RLF	E1748G	chr1:40705617	A/G	0.00	13.94	63.89
CP		MLXIPL	R552L	chr7:73011211	C/A	0.00	23.39	46.15
CP		GTPBP1	A311S	chr:39132191	G/A	0.00	0.00	45.45
CP	CMLPh+022	FRMD4B	P129T	chr3:69351525	G/T	0.00	0.00	44.32
CP		NLGN4X	G503S	chrX:5821212	C/T	1.00	2.57	42.86
CP		OR5B17	P235L	chr11:58125839	G/A	0.00	0.00	35.09
CP		NCAM1	V100I	chr11:113075208	G/A	5.75	32.08	56.32
CP		ACSM4	V119M	chr12:7459282	G/A	0.00	0.00	48.28
CP		FAM124A	R368Q	chr13:51854746	G/A	0.00	0.00	40.63
CP		NALCN	R297C	chr13:101944628	G/A	1.00	4.94	52.00

CP		CARD14	R152Q	chr17:78157817	G/A	0.00	26.17	59.46
CP		SSTR4	R251M	chr20:23016872	G/T	0.00	42.97	52.38
CP		SLC12A5	R231H	chr20:44669091	G/A	1.00	22.07	52.83
CP		FBN3	R233C	chr19:8206866	G/A	0.00	39.60	57.14
CP	CMLPh+023	SLC6A17	W206S	chr1:110717446	G/C	0.00	0.00	40.18
CP		PCDH1	S1147N	chr5:141233881	C/T	0.00	10.65	46.77
CP		KAT6A	G1549S	chr8:41791093	C/T	3.50	74.91	42.69
CP		PATE2	T47I	chr11:125647834	G/A	0.00	0.00	42.03
CP		RECQL	G375V	chr12:21628494	C/A	1.00	52.85	34.33
CP		EXOC3L4	R125S	chr14:103566702	C/A	0.00	0.00	36.36
CP	CMLPh+024	SCNM1	Y41C	chr1:151139612	A/G	0.00	0.00	49.21
CP		ESRRG	T91N	chr1:216850549	G/T	1.00	30.17	45.00
CP		PCM1	N1071K	chr8:17823974	T/A	3.00	35.04	46.67
CP		TMC1	L216F	chr9:75369705	C/T	0.00	5.41	40.88
CP		OR10A5	D181E	chr11:6867456	C/G	0.00	0.00	45.16
CP		SERPINB2	G179S	chr18:61565078	G/A	0.00	47.06	56.00

BC	CML001BC	RTP2	A190V	chr3:187416395	C/T	0.00	10.22	41.38
BC		KCNH3	A314V	chr12:49937815	C/T	0.50	5.61	54.90
BC	CML002BC	FAT4	R1698W	chr4:126242658	C/T	1.00	59.05	53.25
BC		FUT3	R354C	chr19:5843791	C/T	1.00	30.70	38.36
BC		RUNX1	K194N	chr21:36231802	A/T	6.50	72.97	67.50
BC	CML003BC	UBE2A	D84V	chrX:118717100	A/T	1.50	25.66	93.33
BC		ABL1	F486S	chr9:133755488	T/C	7.00	85.77	50.00
BC		SMARCA4	A945T	chr19:11132617	G/A	8.00	49.10	55.00
BC	CML004BC	AMER3	R709His	chr2:131521771	G/A	0.50	34.16	40.91
BC		LAMA2	P1025S	chr6:129621916	C/T	1.75	10.79	45.00
BC		SMC5	*1102Lys	chr9:72967245	T/A	0.00	24.95	48.78
BC		GRIN3A	R1024*	chr9:104335734	C/T	1.00	5.82	40.24
BC		PTPN11	G503V	chr12:112926888	G/T	5.50	41.11	27.27
BC		MESDC2	E130_fs	chr15:81274347	C/-CT	0.00	28.07	45.61
BC		CCDC40	S17L	chr17:78011942	C/T	0.00	8.89	34.10

BC	CML005BC	-	-	-	-	-	-	-
BC	CML006BC	NRAS	Q61R	chr1:115256529	A/G	7.50	81.50	32.73
BC		IKZF1	N159S	chr7:50450292	A/G	2.00	72.59	41.38
BC		CASK	K250R	chrX:41519774	A/G	2.00	10.00	47.45
BC		AK8	R125H	chr9:135730272	G/A	0.00	18.39	65.22
BC		DEFB119	R42H	chr20:29976970	G/A	0.00		46.97
BC	CML007BC	PCLO	L4342W	chr7:82544277	T/G	0.50	8.00	32.35
BC	CML009BC	PPT1	V168A	chr1:40557018	T/C	1.00	8.07	41.38
BC		MDH1B	A272T	chr2:207619829	G/A	0.00		47.31
BC		GPR98	R1745C	chr5:89971182	C/T	0.50	5.37	47.06
BC		CEL	E216Q	chr9:135942015	G/C	1.00	18.92	44.26
BC		LRP4	D449N	chr11:46916335	G/A	0.00	11.94	55.00
BC		CYP2B6	R145W	chr19:41510300	C/T	2.25	15.80	39.69
BC		BCR	F615V	chr22:23610685	T/G	4.50	80.87	44.30
BC	B_1	ASXL1	G641_fs	chr20:31022441	-/G	0.25	77.62	37.00
BC		EPB41L3	P963L	chr18:5396285	G/A	1.25	76.40	37.00

BC		FGFR4	V262M	chr5:176519378	G/A	5.25	45.63	39.00
BC		UBE2A	I33M	chrX:118708918	A/G	1.50	25.66	39.00
BC	B_2	KRT7	R339W	chr12:52639298	C/T	1.25	70.52	40.81
BC		MUDENG	D289N	chr14:57747057	G/A	0.00	20.44	38.31

Suppl. Tab. 5. Somatic mutations identified in 10 BC CML patients by whole exome sequencing and prioritized according to their OncoScore. A yellow box highlights the top 10 OncoScore variants. Red boxes identify genes known to be associated with CML progression. * indicates a stop codon. Reference human genome is GRCh37 (hg19). Variant positions are reported using a +1 notation.

Patient	Gene	Coordinates	Mutation	OncoScore
CML003BC	ABL1	chr9:133755488	F486S	85.77
CML006BC	NRAS	chr1:115256529	Q61R	81.50
CML009BC	BCR	chr22:23610685	F615V	80.87
BRAN_1	ASXL1	chr20:31022441	G641_fs	77.62
BRAN_1	EPB41L3	chr18:5396285	P963L	76.40
CML002BC	RUNX1	chr21:36231802	K194N	72.97
CML006BC	IKZF1	chr7:50450292	N159S	72.59
BRAN_2	KRT7	chr12:52639298	R339W	70.52
CML002BC	FAT4	chr4:126242658	R1698W	59.05
CML003BC	SMARCA4	chr19:11132617	A945T	49.10
BRAN_1	FGFR4	chr5:176519378	V262M	45.63
CML004BC	PTPN11	chr12:112926888	G503V	41.11
CML004BC	AMER3	chr2:131521771	R709His	34.16
CML002BC	FUT3	chr19:5843791	R354C	30.70
CML004BC	MESDC2	chr15:81274347	E130_fs	28.07
CML003BC	UBE2A	chrX:118717100	D84V	25.66
BRAN_1	UBE2A	chrX:118708918	I33M	25.66
CML004BC	SMC5	chr9:72967245	*1102Lys	24.95
BRAN_2	MUDENG	chr14:57747057	D289N	20.44
CML009BC	CEL	chr9:135942015	E216Q	18.92
CML006BC	AK8	chr9:135730272	R125H	18.39
CML009BC	CYP2B6	chr19:41510300	R145W	15.80
CML009BC	LRP4	chr11:46916335	D449N	11.94
CML004BC	LAMA2	chr6:129621916	P1025S	10.79
CML001BC	RTP2	chr3:187416395	A190V	10.22
CML006BC	CASK	chrX:41519774	K250R	10.00
CML004BC	CCDC40	chr17:78011942	S17L	8.89
CML009BC	PPT1	chr1:40557018	V168A	8.07

CML007BC	PCLO	chr7:82544277	L4342W	8.00
CML004BC	GRIN3A	chr9:104335734	R1024*	5.82
CML001BC	KCNH3	chr12:49937815	A314V	5.61
CML009BC	GPR98	chr5:89971182	R1745C	5.37
CML006BC	DEFB119	chr20:29976970	R42H	0.00
CML009BC	MDH1B	chr2:207619829	A272T	0.00

Suppl. Tab. 6. Chromosomal regions recurrently deleted in prostate cancer and associated OncoScore. Genes scoring more than 90th percentile are highlighted in yellow.

8p21

GeneList Sorted	OncoScore
TNFRSF10B	86.32231
TNFRSF10C	85.93896
TNFRSF10A	85.80654
TNFRSF10D	82.99415
LZTS1	80.58777
NKX3-1	75.53011
BNIP3L	72.25596
RHOBTB2	71.41589
CCAR2	71.09352
PNMA2	61.31472
PEBP4	59.1257
PPP2R2A	57.81989
EXTL3	56.13228
PDLIM2	51.97434
DOK2	51.84372
LOXL2	49.22038
ADAM28	45.99757
CHMP7	45.54588
SLC25A37	44.64252
CDCA2	44.43232
ZNF395	42.13431
XPO7	36.78816
HMBOX1	36.77761
TRIM35	36.05285

NEFM	34.58798
PIWIL2	34.41141
STC1	34.40522
FZD3	33.86296
CLU	30.76347
NEFL	30.51222
CSGALNACT1	28.06776
SH2D4A	27.9588
EGR3	26.45031
ADAMDEC1	25.74433
INTS10	25
PHYHIP	25
CCDC25	25
POLR3D	24.60468
LGI3	24.14498
SCARA3	24.14498
PBK	23.71828
SCARA5	23.17212
BIN3	23.05865
DOCK5	22.5
ELP3	21.65362
SLC18A1	21.39096
ADAM7	21.15699
PTK2B	20.45158
HR	20.31993
NPM2	19.43661
SORBS3	18.02643
ESCO2	17.73867
FGF17	17.24926
EPHX2	16.72884
SLC39A14	16.54061
BMP1	14.71257
SFTPC	14.35766

DPYSL2	13.88642
NKX2-6	13.0316
MIR320A	12.5
LOC389641	12.5
DMTN	12.30234
REEP4	12.30234
LPL	12.11658
STMN4	12.1116
ATP6V1B2	11.52933
GFRA2	10.69021
KCTD9	9.19704
EBF2	8.033926
GNRH1	7.887744
PPP3CC	6.390384
PNOC	6.390384
CHRNA2	5.286983
ADRA1A	2.607558
LOC100128993	0
LZTS1-AS1	0
LOC102467222	0
LOC286114	0
LOC101929172	0
NUDT18	0
LOC100507071	0
C8orf58	0
BIN3-IT1	0
LOC101929237	0
LOC286059	0
LOC254896	0
R3HCC1	0
LOC100507156	0
ENTPD4	0
LOC101929294	0

LOC101929315	0
MIR6841	0
MIR6876	0
MIR548H4	0
MIR6842	0
MIR6843	0
MIR3622B	0
MIR3622A	0
MIR4287	0
NUGGC	0
MIR4288	0
MIR7641-2.	0
EXTL3-AS1	0
INTS9	0

10q23

GeneList Sorted	OncoScore
PTEN	74.08989
KLLN	72.10571
SNCG	71.67795
TNKS2	57.62342
CEP55	57.24303
PLCE1	57.2149
HELLS	50.57924
KIF11	50.01044
MMRN2	50
KIF20B	45.61058
MINPP1	44.90842
FAS	40.57179
IFIT5	39.81025
MIR346	36.90702
NUTM2A	36.90702
ANKRD22	34.15941
TSPAN14	33.33333
BMPR1A	33.21629
IFIT2	32.23493
WAPL	31.65251
NRG3	31.33969
ADIRF	30.65736
IFIT3	30.34195
ACTA2	29.83578
IFIT1	29.18016
CYP26A1	28.95384

MIR107	27.9588
PDLIM1	27.38459
LGI1	27.16741
SLC16A12	26.33395
GHITM	25
HOST2	25
FGFBP3	25
EXOC6	25
C10orf99	24.60468
PAPSS2	23.94431
MARCH5	23.05865
MYOF	21.81099
HHEX	21.68591
STAMBPL1	20.9691
NOC3L	20.9691
PPP1R3C	19.38138
LIPA	19.24954
IDE	19.19956
CH25H	18.17582
CYP2C8	17.3864
ANKRD1	17.26214
CPEB3	15.51526
RBP4	14.55853
PANK1	14.0625
CYP26C1	13.8007
RPP30	12.72308
RNLS	12.66979
CYP2C18	11.13807
CYP2C9	10.27637
PCGF5	9.19704
CYP2C19	8.629592
FFAR4	8.485558
HECTD2	8.333333

GLUD1	7.565413
LIPF	7.391112
GRID1	7.355521
BTAF1	7.355521
HTR7	7.121369
PDE6C	7.033969
LDB3	4.554167
RGR	4.198814
OPN4	4.159346
LIPN	3.843109
DYDC1	0
LOC101929574	0
LOC102723703	0
SH2D4B	0
NRG3-AS1	0
LOC105378397	0
CDHR1	0
LRIT2	0
LRIT1	0
LINC00858	0
CCSER2	0
LINC01519	0
LOC101929646	0
LOC101929662	0
LINC01520	0
GRID1-AS1	0
FAM25A	0
FAM35A	0
NUTM2A-AS1	0
LINC00863	0
NUTM2D	0
LINC00864	0
MIR4678	0

ATAD1	0
CFL1P1	0
LIPJ	0
LIPK	0
LIPM	0
ACTA2-AS1	0
FAS-AS1	0
MIR4679-2	0
MIR4679-1	0
IFIT1B	0
SLC16A12-AS1	0
FLJ37201	0
LINC00865	0
LINC01375	0
LOC101926942	0
LOC105378430	0
LINC00502	0
HECTD2-AS1	0
TNKS2-AS1	0
MARK2P9	0
FRA10AC1	0
PIPSL	0
PLCE1-AS2	0
PLCE1-AS1	0
TBC1D12	0
ACSM6	0

12p13

GeneList Sorted	OncoScore
ETV6	86.66713
TNFRSF1A	82.75696
ING4	82.64462
CDKN1B	76.17695
MIR200C	73.60375
LTBR	73.58116
FOXM1	72.67542
GPRC5A	67.87925
KDM5A	65.75595
TIGAR	65.65343
PRB2	64.7568
CCND2	60.05958
RAD51AP1	59.21765
KLRK1	59.07163
MIR141	56.33829
STYK1	56.25
MLF2	55.9176
ZNF384	54.90243
BCL2L14	54.5022
NOP2	52.58137
ATF7IP	48.72589
APOLD1	45.54588
EMP1	45.34893
GPRC5D	45.24133
CD9	42.009

CREBL2	41.9382
B4GALNT3	41.66667
FBXL14	41.66667
CHD4	41.47205
NANOG	40.50757
LAG3	39.60822
PTPN6	39.20466
CD27	38.48728
PLEKHG6	37.5
CD163	37.36106
CRACR2A	36.78816
DDX47	36.78816
KLRD1	36.28486
AICDA	36.22575
DUSP16	35.91646
ACRBP	35.59997
HEBP1	35.16956
PHB2	34.53653
KLRC1	33.92152
FKBP4	33.80231
RBP5	33.33333
KLRC2	32.75394
ERC1	32.24905
TEAD4	31.97303
WNT5B	31.68285
CLEC4C	31.49009
LRP6	31.28018
KLRB1	31.09263
CLEC2B	30.86642
CLEC2A	30.65736
PHC1	30.45923
FOXJ2	30.279
CDCA3	30.04404

LPAR5	29.82179
MFAP5	29.56285
USP5	29.10563
CD69	28.80861
CLEC9A	28.6693
CLEC1B	27.9588
KLRG1	27.89746
COPS7A	27.59112
YBX3	27.59112
FGF6	27.49172
GDF3	27.14019
KLRF1	27.0934
CLEC2D	26.88579
GABARAPL1	25.24664
A2ML1	24.8014
MAGOHB	24.60468
CLEC12A	24.2232
SLC2A14	24.03524
RAD52	23.74517
M6PR	23.71209
PRMT8	23.39822
CD4	23.26066
GAPDH	23.21336
ADIPOR2	22.88884
GALNT8	22.77294
APOBEC1	22.65601
KLRC3	22.5
CLEC4A	20.70105
SLC2A3	20.66493
ENO2	20.64187
P3H3	20.43824
C1RL	20.43824
NDUFA9	19.43661

GPR19	19.38914
NCAPD2	18.75
DPPA3	18.64233
TSPAN9	18.39408
TPI1	18.24837
PTMS	18.14978
TULP3	17.63082
CLEC4D	17.5
TAPBPL	16.66667
ATN1	16.54138
A2M	16.31668
EMG1	16.28949
KLRC4	16.09665
KCNA6	15.1395
FGF23	14.12723
ANO2	13.21746
OLR1	13.07967
MRPL51	12.5
C12orf4	12.30234
AKAP3	12.2123
C3AR1	12.1116
CLSTN3	12.01762
SCNN1A	11.90721
C1R	11.8873
CD163L1	11.38647
CLECL1	10.95384
PARP11	10.21912
GSG1	10.21912
VWF	9.838036
CLEC4E	9.773696
CLEC12B	9.19704
PZP	8.864033
WNK1	8.71856

NTF3	8.177576
CLEC7A	7.736353
DYRK4	7.605946
GRIN2B	7.145091
C1S	7.13981
VAMP1	6.929157
GNB3	6.902479
KCNA1	6.891921
SPSB2	6.463047
CACNA1C	6.379202
LPCAT3	6.0558
KCNA5	5.789721
CACNA2D4	4.96028
SLC6A13	4.757163
SLC6A12	3.878814
PEX5	3.809764
NINJ2	3.138647
LOC100288778	0
FAM138D	0
IQSEC3	0
LOC101929384	0
LOC102723544	0
LOC105369595	0
LOC100049716	0
MIR3649	0
LRTM2	0
LINC00940	0
DCP1B	0
CACNA1C-IT2	0
CACNA1C-AS4	0
CACNA1C-IT3	0
CACNA1C-AS2	0
CACNA1C-AS1	0

LOC283440	0
LOC100507424	0
THCAT155	0
CCND2-AS1	0
LOC101929549	0
LOC101929584	0
CD27-AS1	0
SCARNA10	0
SCARNA11	0
PIANP	0
GPR162	0
LOC105369632	0
DSTNP2	0
C12orf57	0
RNU7-1	0
LOC105369635	0
SCARNA12	0
C1RL-AS1	0
ACSM4	0
NECAP1	0
POU5F1P3	0
ZNF705A	0
FAM90A1	0
FAM86FP	0
LOC101927905	0
LINC00937	0
CLEC6A	0
RIMKLB	0
LINC00612	0
A2M-AS1	0
A2MP1	0
MIR1244-2	0
MIR1244-3	0

MIR1244-1	0
MIR1244-4	0
LINC00987	0
LOC642846	0
LOC101930452	0
LOC101928030	0
DDX12P	0
LOC374443	0
KLRF2	0
LOC100506159	0
LOC400002	0
LOC102724020	0
TMEM52B	0
LOC101928100	0
KLRC4-KLRK1	0
KLRA1P	0
TAS2R7	0
TAS2R8	0
TAS2R9	0
TAS2R10	0
PRR4	0
PRH1-PRR4	0
PRH1	0
TAS2R13	0
PRH2	0
PRH1-TAS2R14	0
TAS2R14	0
TAS2R50	0
TAS2R20	0
TAS2R19	0
TAS2R31	0
TAS2R46	0

TAS2R43	0
TAS2R30	0
SMIM10L1	0
TAS2R42	0
PRB3	0
PRB4	0
PRB1	0
LINC01252	0
MANSC1	0
BORCS5	0
RPL13AP20	0
MIR614	0
LOC100506314	0
HTR7P1	0
FAM234B	0
MIR7641-2	0
PLBD1	0
PLBD1-AS1	0
